# The Effect of Social Support Features and Gamification on a Web-Based Intervention for Rheumatoid Arthritis Patients: Randomized Controlled Trial

**DOI:** 10.2196/jmir.3510

**Published:** 2015-01-09

**Authors:** Ahmed Allam, Zlatina Kostova, Kent Nakamoto, Peter Johannes Schulz

**Affiliations:** ^1^Institute of Communication and HealthFaculty of Communication SciencesUniversity of Lugano (Università della Svizzera italiana)LuganoSwitzerland; ^2^Marketing DepartmentPamplin College of BusinessVirginia TechBlacksburg, VAUnited States

**Keywords:** social support, gaming, experimental games, eHealth, rheumatoid arthritis, randomized controlled trial, multilevel analysis, patient empowerment, physical activity, health care utilization

## Abstract

**Background:**

Rheumatoid arthritis (RA) is chronic systematic disease that affects people during the most productive period of their lives. Web-based health interventions have been effective in many studies; however, there is little evidence and few studies showing the effectiveness of online social support and especially gamification on patients’ behavioral and health outcomes.

**Objective:**

The aim of this study was to look into the effects of a Web-based intervention that included online social support features and gamification on physical activity, health care utilization, medication overuse, empowerment, and RA knowledge of RA patients. The effect of gamification on website use was also investigated.

**Methods:**

We conducted a 5-arm parallel randomized controlled trial for RA patients in Ticino (Italian-speaking part of Switzerland). A total of 157 patients were recruited through brochures left with physicians and were randomly allocated to 1 of 4 experimental conditions with different types of access to online social support and gamification features and a control group that had no access to the website. Data were collected at 3 time points through questionnaires at baseline, posttest 2 months later, and at follow-up after another 2 months. Primary outcomes were physical activity, health care utilization, and medication overuse; secondary outcomes included empowerment and RA knowledge. All outcomes were self-reported. Intention-to-treat analysis was followed and multilevel linear mixed models were used to study the change of outcomes over time.

**Results:**

The best-fit multilevel models (growth curve models) that described the change in the primary outcomes over the course of the intervention included time and empowerment as time-variant predictors. The growth curve analyses of experimental conditions were compared to the control group. Physical activity increased over time for patients having access to social support sections plus gaming (unstandardized beta coefficient [B]=3.39, *P*=.02). Health care utilization showed a significant decrease for patients accessing social support features (B=–0.41, *P*=.01) and patients accessing both social support features and gaming (B=–0.33, *P*=.03). Patients who had access to either social support sections or the gaming experience of the website gained more empowerment (B=2.59, *P*=.03; B=2.29, *P*=.05; respectively). Patients who were offered a gamified experience used the website more often than the ones without gaming (*t*_91_=–2.41, *P*=.02; *U*=812, *P*=.02).

**Conclusions:**

The Web-based intervention had a positive impact (more desirable outcomes) on intervention groups compared to the control group. Social support sections on the website decreased health care utilization and medication overuse and increased empowerment. Gamification alone or with social support increased physical activity and empowerment and decreased health care utilization. This study provides evidence demonstrating the potential positive effect of gamification and online social support on health and behavioral outcomes.

**Trial Registration:**

International Standard Randomized Controlled Trial Number (ISRCTN): 57366516; http://www.controlled-trials. com/ISRCTN57366516 (Archived by webcite at http://www.webcitation.org/6PBvvAvvV).

## Introduction

### Background

Rheumatoid arthritis (RA) is a chronic systemic disease that affects the joints, connective tissues, muscles, tendons, and fibrous tissue [1]. The disease predominantly targets adults aged between 20 and 40 years and it is more prevalent in women than in men [1]. Because RA affects people during an especially productive economic period of their lives, it is considered a serious public health problem globally. In Switzerland, up to 1% of the population suffers from RA. Its estimated cost to Swiss society reaches €23,982 per patient a year [2]. The impact of RA goes beyond physical and economic aspects; it affects patients psychologically and emotionally, making them suffer severe consequences and losses [3].

The fast adoption and use of the Internet during the last decade and the proliferation of Web-based apps (eg, wikis, blogs, forums, chat rooms, social networking, and video sharing) with the emergence of Web 2.0 has made health care providers realize the importance and potentials of the medium to target patients [4]. As Internet penetration has been increasing—the share of the population using the Internet was estimated to be 63.2% in Europe and 78.6% in North America as of June 2012 [5]—the Internet has become a necessity and part of people’s daily life. In April 2012, 82% of all American adults older than 18 years used the Internet or email at least occasionally [6] and 70% of Internet users aged 65 and older used the Internet on a typical day.

The health care domain, like many other fields, benefits from this technological advancement; people tend to seek online health information, virtual communities, and Web-based apps, and many other forms of online health services have started to emerge to satisfy people’s needs.

The basic idea behind informative eHealth offers issued by official or medical institutions is to help patients better cope with chronic conditions, primarily by providing correct and up-to-date information [7]. The goals include knowledge gain, better health-related quality of life, behavior change beneficial to patients’ health, and less unnecessary utilization of the health system.

To attain these goals, several interventions and self-management programs were developed and their efficacy for helping patients to achieve these goals tested. Many of these interventions emphasized empowerment and self-management [8-13]. The aim of empowering patients is to enable them to use the health information they acquire for making decisions and judgments that help them manage their disease. The effectiveness of more physical activity [14-16] and self-management [17-19] has been long established in treating arthritis-related pain and disabilities. One of the first Internet-based interventions was created and studied by Van Den Berg and colleagues [20] to increase physical activity of patients with RA. They compared 2 versions of an Internet-based intervention; the first included a physical activity program with individual/tailored guidance, a bicycle ergometer, and group contacts, and the second consisted of only general information on exercises and physical activity. They found that the proportion of RA patients who reported meeting the physical activity recommendations was significantly higher in the first program than in the second.

Along the same lines, another experiment done by Lorig and colleagues [9] tested the efficacy of delivering their developed arthritis self-management program through an Internet-based intervention. The intervention improved health status measures at 1 year, presenting an alternative to the conventional small-group arthritis self-management program.

In addition to the provision of information, the Internet has more to offer to attain these goals; in particular, health websites can be designed to provide social support to their users. Social support is an important factor because it has been shown that seeking and receiving assistance from other people is vital to chronically ill persons and it is associated with an increase in empowerment and self-management skills [21-24]. Consequently, the online health communities providing online social support become an essential resource for patients searching for others with similar health concerns [25].

Two forms of social support can be distinguished: structural support (availability of support givers) and functional support (perception of support) [26]. Both can help chronically ill patients to cope emotionally and practically [27,28]. There is strong empirical evidence that the support patients receive from their social environment can help them face the challenges and improve their self-management of chronic disease [29]. Our focus, as in most of the research, is on the structural support that usually comes from family, friends, and significant others [30-31], but we shift the perspective to structural social support delivered online by other website users, be they experts, physicians, or other patients.

Many studies, predominantly qualitative studies, analyzed post comments and messages published on bulletin boards or forums for patients with chronic diseases [32-37] and categorized the types and themes of online social support. There is no consensus on the order or importance of types of support, but a recurring categorization across studies distinguishes informational, emotional, and practical (or instrumental) support [38].

Literature reviews, such as Eysenbach et al [21] and more recently Griffiths et al [39], investigated the evidence of an effect of online peer-to-peer interactions on users/patients and Internet support groups (ISGs) in the area of depressive symptoms. According to Eysenbach et al [21], virtual communities cannot harm people; however, there was no evidence of benefit either, which suggests more research is needed to understand for whom and under which conditions social support could work [21]. For the Griffiths et al review [39], there was little high-quality evidence dealing with the efficacy of ISGs on coping with depression, suggesting a need for high-quality randomized controlled trials (RCTs) in this domain.

The concept of “gamification” has emerged recently. It is described as the application of game design elements in a nongame context to motivate or influence participation [40-42] and sometimes also refers to designing new serious games [43]. Significant knowledge increase by, and high users’ appreciation of, gamified apps are shown in studies on gamifying laboratory experience for undergraduate microbiology students [44], evaluating a 3D serious game for advanced life support retraining [45], and major incident triage training [46]. However, much of the evidence for an influence of gamification on people’s mind and behavior is anecdotal, with only 1 very recent systematic literature review [47] as discussed by Cugelman [48]. The review included 24 studies for final evaluation, which examined the relationship between motivation affordances (eg, points, badges, leaderboards, reward) and behavioral (use of the system/application and intentions surveyed through questionnaires) and psychological outcomes (motivation, attitude, and enjoyment) using evaluative interviews or questionnaires [47]. Only 1 study [43] from the review of Hamari el al [47] used validated psychometric measurements and its context was health/exercise. The predominant context of gamification studies was education or learning and their participants were students [41-44] or they were conducted in crowdsourcing systems (ie, Amazon Mturk) [47].

Do social support features and gamification elements affect the attainment of the goal of health websites, provided they do have effects? Given the dearth of hard evidence for the effect of gamification and a conflicted or at most modest evidence for the effect of online social support of Web-based health interventions on health outcomes, we decided to conduct an experimental study that included both. The intervention used in this study is ONESELF, a Web-based intervention designed and operated for chronically ill patients with RA [49].

ONESELF has informational and online support features and a gamified user experience, but access to these features was manipulated for the different experimental groups. We developed ONESELF in collaboration with the doctors of the Swiss Rheumatology Association. The ONESELF website is compliant with the Health on the Net (HON) Foundation guidelines and is HON certified.

### ONESELF Overview

ONESELF began in late 2004 (early prototype and development) as a project to test the efficacy of an online tool (using the Internet as a medium) to enhance the self-management of chronic low back pain patients in the Italian-speaking population of Switzerland [50]. The results of the pilot study (June 2004 to June 2005) were promising; the patients’ experiences and feedback obtained helped to improve and redesign the website. As a result, another study [51] with a larger sample (129 patients who filled in pre- and postuse questionnaires) was conducted between June 2006 and May 2007. The results confirmed the pilot study with patients reporting decreased painkiller intake and an increase in knowledge of back pain. Moreover, there was an acknowledgment of the benefits for improving the communication with doctors and family and colleagues. The qualitative analysis done in the same study [51] and in another by Zufferey et al [52] reported that patients experienced positive effects on self-management attitudes and behaviors with regard to their chronic low back pain condition. The design principles followed in developing ONESELF content (patient centeredness and rich information), monitoring by health professionals, and providing a tailored experience to patients proved to be a promising, viable tool for helping patients enhance and develop their self-management of chronic low back pain. This was true especially for patients who were engaged in the process of self-management or were inclined to self-manage [51,52].

The research on ONESELF was extended to target other chronic patients when another release of ONESELF took place in June 2008, which included a new section for patients with fibromyalgia syndrome. A first cross-sectional study took place in the same period, for which 209 patients were recruited to evaluate the effectiveness of the Internet-based education intervention built on principles of personalization and participatory design [53]. An a priori model was tested that included the patients’ self-reported use of the website, health knowledge, self-management behavior, and health outcomes. The results showed that using the tailored functionalities that were the result of end users’ involvement and participation in the design and development process improved patients’ health knowledge and, in turn, improved the self-management skills that can decrease the effects of fibromyalgia syndrome and eventually lead to reaching better health outcomes. The online gymnasium for patients with fibromyalgia syndrome [54] was 1 of the many examples of personalization and tailoring done on ONESELF [53]. A final experiment was conducted on ONESELF before its most recent version was released [55]. The goal was to investigate and test the role of functional interactivity on patients’ knowledge, empowerment, and health outcomes. It was a pretest-posttest experimental design in which 165 patients suffering from fibromyalgia syndrome were randomly allocated to 3 groups corresponding to different levels of functional interactivity. The study reported a model-driven evaluation that tested whether health knowledge and empowerment mediated a possible relationship between the availability of interactive features and individuals’ health outcomes. Functional interactivity did not affect empowerment or knowledge; however, knowledge and some dimensions of the empowerment positively affected the health outcomes.

### Research Aims

Because of the importance of the impact of knowledge and empowerment on health outcomes [55], the goal in this study was to target these constructs and enhance their effects. Inspired by the model-driven approach presented by Camerini and Schulz [55], the current RCT aimed at testing the new experimental manipulation (online social support and gamification) in a similar approach. The primary outcomes studied were patients’ physical activity, health care utilization, and prescription medication overuse. Moreover, we considered secondary outcomes (empowerment, knowledge), which were also included as predictors of the primary ones. To our knowledge, this is 1 of the first RCT studies that includes gamification as part of experimental manipulation and studies its effect on cognitive and behavioral outcomes of patients diagnosed with RA.

The main part of this study looks into the effects of social support features and gamification on the primary outcomes, expecting beneficial effects (more exercise, less health care utilization, less medication overuse) for the former, and treating the direction of an effect of gamification as an open research question. In a side analysis, the effect of gamification on website use will be addressed.

## Methods

### Overview

The study was conducted as an RCT experiment with RA patients in Ticino (Italian-speaking part of Switzerland). Data (assessment of the intervention) were collected from the last week of February 2013 until July 2013.

A new independent section about RA was created and added to the other 2 sections of the ONESELF website, which were completely rewritten in Drupal [56]. Drupal was chosen because of its flexibility and scalability to develop features and functionalities through writing custom modules that would integrate with the other core modules available from the system. For this study, multiple custom modules were written that took care of the experimental manipulation, the gamification of many of the functionalities of the website, and many other tailored features specifically for the conducted experiment as described in the following sections. Moreover, it is a well-documented open source content management system (CMS) that has a vibrant and active development community making it a reliable CMS.

**Figure 1:**
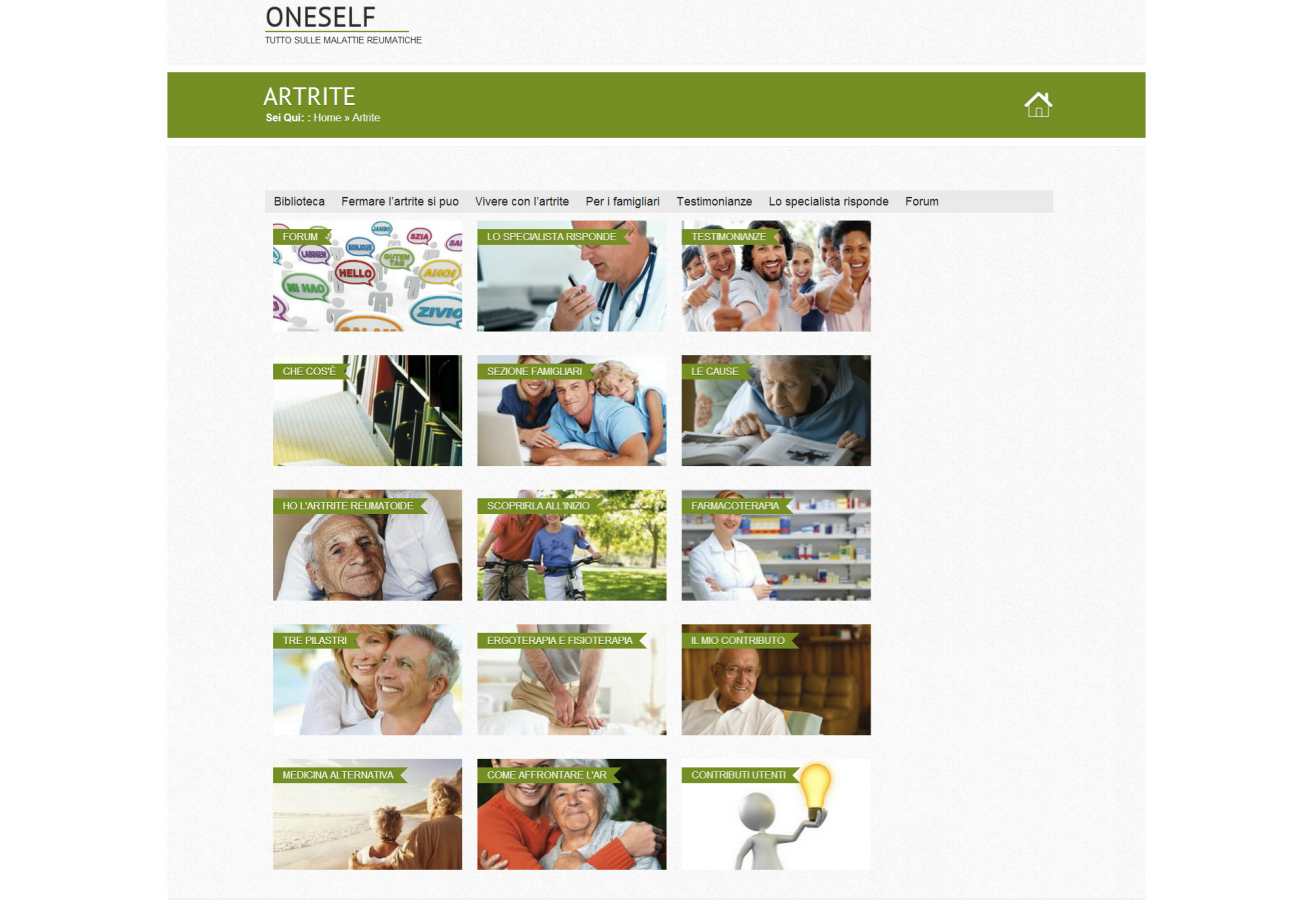
Screenshot of the complete rheumatoid arthritis section on the ONESELF website.

The single-blinded experiment tested, in a repeated-measure design, the effect of website sections and features offering social support and a corresponding gaming experience compared to a standalone informative version of the website. Participants were randomly allocated to the parallel experimental groups, unaware of any manipulation, and blinded to one other. Each group had access to different sections and features of the website, including a control group with no access at all. Participants filled in questionnaires at 3 occasions: baseline, posttest 2 months later, and a follow-up after another 2 months. The study was approved by the Ethical Committee of Canton Ticino (the Italian-speaking part of Switzerland). Figure 1 presents the complete RA section of the website.

### Website Sections and Features

The original purpose of ONESELF was to provide information that promotes good health outcomes. Building on the past and learned experiences in developing the ONESELF website and its content and following the principle of patient centeredness, patients’ needs were translated into material and content prepared with the help of health professionals, providing the right reading level, in the right language, using culturally appropriate images [51]. ONESELF was designed to target the declarative knowledge, the procedural knowledge, and the integration of knowledge toward a behavioral response [51].

**Figure 2:**
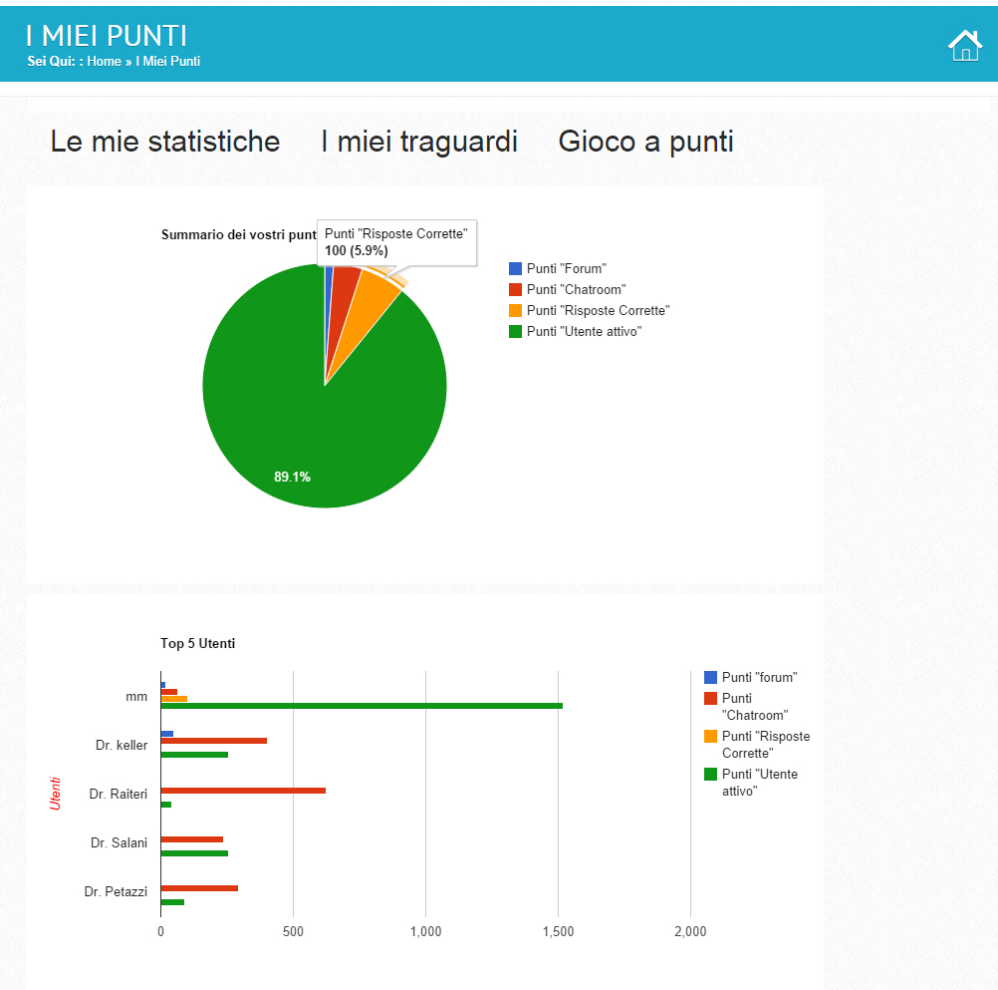
Screenshot of My Points section of the ONESELF website displaying statistics of the collected points for every action in each category and leadership board of top 5 users.

**Figure 3:**
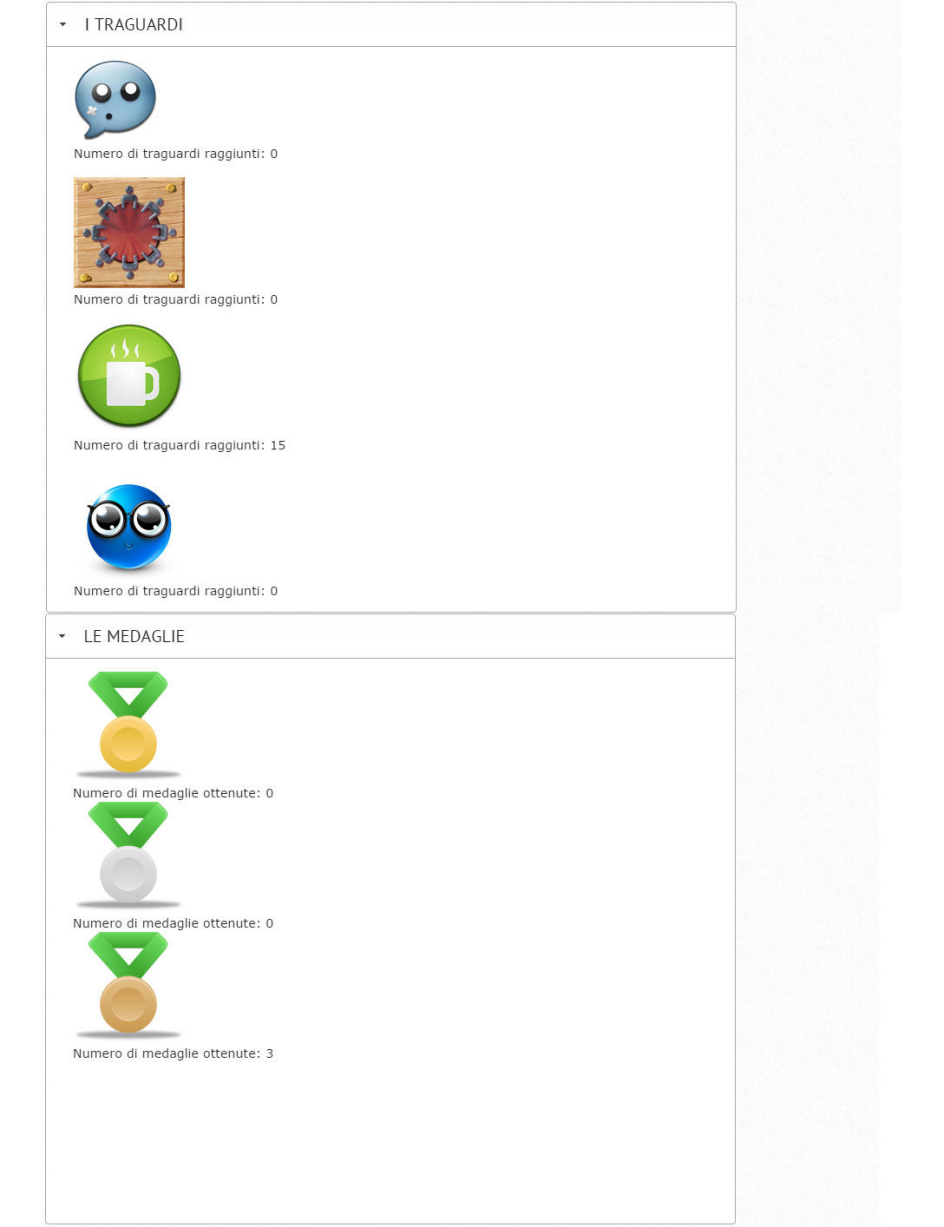
Screenshot of My Points section of the ONESELF website displaying the badges and medals achieved during the intervention.

The main sections and features were:

1. One section included informative webpages about RA and served to improve the declarative knowledge of the patients by using simple layperson language to present and describe arthritis, covering its main aspects and the issues around it.
2. Three sections included articles and videos prepared in collaboration with physiotherapists, ergotherapists, and doctors. They explained methods and techniques that helped in coping with RA especially in one’s everyday life. These sections served to minimize the negative impact of the disease on patients’ lives at home and at the workplace. Treatment options such as medications and alternative therapies were also discussed in these sections. The goal was to target the procedural knowledge of the patients by explaining the steps and actions that contribute to better disease management.
3. The testimonies section included video interviews with patients speaking about their experience with the disease and the way they dealt with it.
4. Another section offered video interviews with doctors about different therapies and the ways for handling the pain and getting over the obstacles presented by RA.
5. A forum and chat room were implemented and made available to the patients. During the course of the intervention, 9 prescheduled sessions were offered in the chat room. Patients were able to see the agenda of the planned sessions and the topic that would be addressed by each doctor. In each session, a different doctor participated in the chat with the patients, moderated by the research team. Patients discussed their questions and concerns with the doctor. The discussion was visible to all participants in the chat room.
6. A patients’ blog was a tool for patients to contribute to the website. They were allowed to write anything and attach files and materials accessible to other patients.
7. Gamification was added to encourage and motivate the patients to use the platform more. Participants’ actions and contributions to the platform were rewarded by points that allowed for collecting different badges and gaining various medals. Points were given according to patients’ contributions and interaction with different features of the website. We differentiated between immediate and delayed rewarding. Points were immediately rewarded for posting, commenting/replying in the forum, writing and publishing in the patients’ blog, and answering 1 of the quizzes correctly that were attached to the different webpages. Delayed rewarding was given for visiting and exploring the different webpages and sections and for participating in the chat room sessions. Points were automatically calculated and distributed at midnight (Swiss time). A section called “My Points” (Figures 2 and 3) was available in which patients could see their rank and statistics for their performance in collecting points, badges, and medals. The same section contained a leadership board that showed the top 5 users from among the same experimental group and gave information about their points collected in the different categories. The rules of the game and the explanation of how to earn points, badges, and medals were included in the same section that announced a real prize for the top 5 users at the end of the intervention.

Sections 1 and 2 offered information primarily, whereas the other sections provided social support to users, including emotional, practical, and informational support from different parties: RA patients for testimonies, physicians and doctors for video interviews, and both for the forum and chat room. For additional information and presentation of different features and sections implemented in the platform, refer to Multimedia Appendix 1.

### Recruitment and Participants

Recruitment of participants lasted from November 2012 until February 2013. Patients were introduced to the experiment through brochures left with health care providers (rheumatologists, physiotherapists, ergotherapists, and psychologists). There was a continuous collaboration between the research team and the health care providers who helped in the recruitment process at their corresponding clinics and rehabilitation centers. In this way, we were assured that prospective participants received a diagnosis of RA from their doctor and had not participated in another experiment or research study before. Moreover, the screening process included predefined inclusion criteria the patients had to meet to participate in the study: (1) received a diagnosis for RA from a doctor, (2) had sufficient competence to use the website effectively (self-reported ability to navigate the Internet and browse websites for health information), (3) did not have any other major chronic illness (eg, cancer, diabetes), (4) had Internet access, (5) was willing to use the website for at least 1 hour per week, and (6) was fluent in the Italian language. If they met these criteria, patients were given a brochure with a brief description of the study and a contact and consent form to be signed and sent back to the research team (see Multimedia Appendix 2).

### Experimental Groups

Patients were randomly allocated to 1 of 4 experimental groups or a control group with no access to the website. The randomization of the patients was based on a computerized random number generator that handled the patients’ assignment to different groups.

One group of patients had access to informational sections only, another group additionally had access to the social support sections but not to the gaming section, a third group had access to the gaming but not the social support sections, and a final group had access to everything. Table 1 illustrates the experimental design.

**Table 1:** Overview of experimental conditions.

Group access	No access to information sections		Access to informational sections	
	No access to social support features	Access to social support features	No access to social support features	Access to social support features
No access to gaming feature	Control group		Information group	Support group
Access to gaming feature			Gaming group	Support plus gaming group

### Procedure

Once patients were randomly allocated to 1 of the experimental groups, a researcher with the help of a student assistant contacted the patients (via email or phone) to inform them of further instructions. They also contacted the patients when questionnaires were ready at later assessment points.

Patients who were to use the ONESELF website were given a brief explanation of the study and provided with further instructions to access the website through generated accounts with unique user IDs. By following the instructions, patients were able to log in to the website and edit their user profile by choosing a new username and password. The first time patients signed in to the platform, they were prompted to fill in an online baseline questionnaire. Access to the website was blocked until the patients finished it. After that, patients were free to explore the different sections and features of the website available to their experimental group. On the patient profiles, a paragraph called “ethical consideration” was clearly visible, which stated that the study was approved by an ethical committee and participation was voluntary and that they could revoke the consensus they had given and quit the study without any need for justification or reason at any time.

During the experiment, the website was updated frequently and we notified the patients continually by email. The updating and notification mechanism allowed for presenting communication messages in modal windows (modal dialogs blocking the interaction with other elements and windows on the website) to the patients when they accessed the website. In addition, they were also redirected to different pages after closing these messages for checking the new published content. Moreover, a ticker was displayed on the different sections of the website showing the latest messages addressing the patients. In addition to sending emails, patients who had access to social support features were also notified by short message service (SMS) text message about the chat room appointments, inviting them to participate. A researcher from the research team played the role of moderator during the chat room sessions in addition to coordinating the interaction and communication on the forum between the patients and invited health professionals throughout the intervention. The researchers and the student assistant mentioned previously did not participate in the data analysis.

Two months after the beginning of the intervention, the posttest was presented again in a modal window and patients had to complete it if they wished to continue using the site. The final follow-up questionnaire was filled in 2 months after the posttest. Patients were contacted by email and/or telephone when the online questionnaire was ready. A maximum of 2 phone calls were made as a reminder to fill in the questionnaires.

For the control group, they were instructed to fill out a paper-and-pencil version of the questionnaire that was sent by mail to their contact address, having been informed that we were interested in collecting general information about RA with the aim of developing an online platform that would be accessible to the public at a later date.

**Figure 4:**
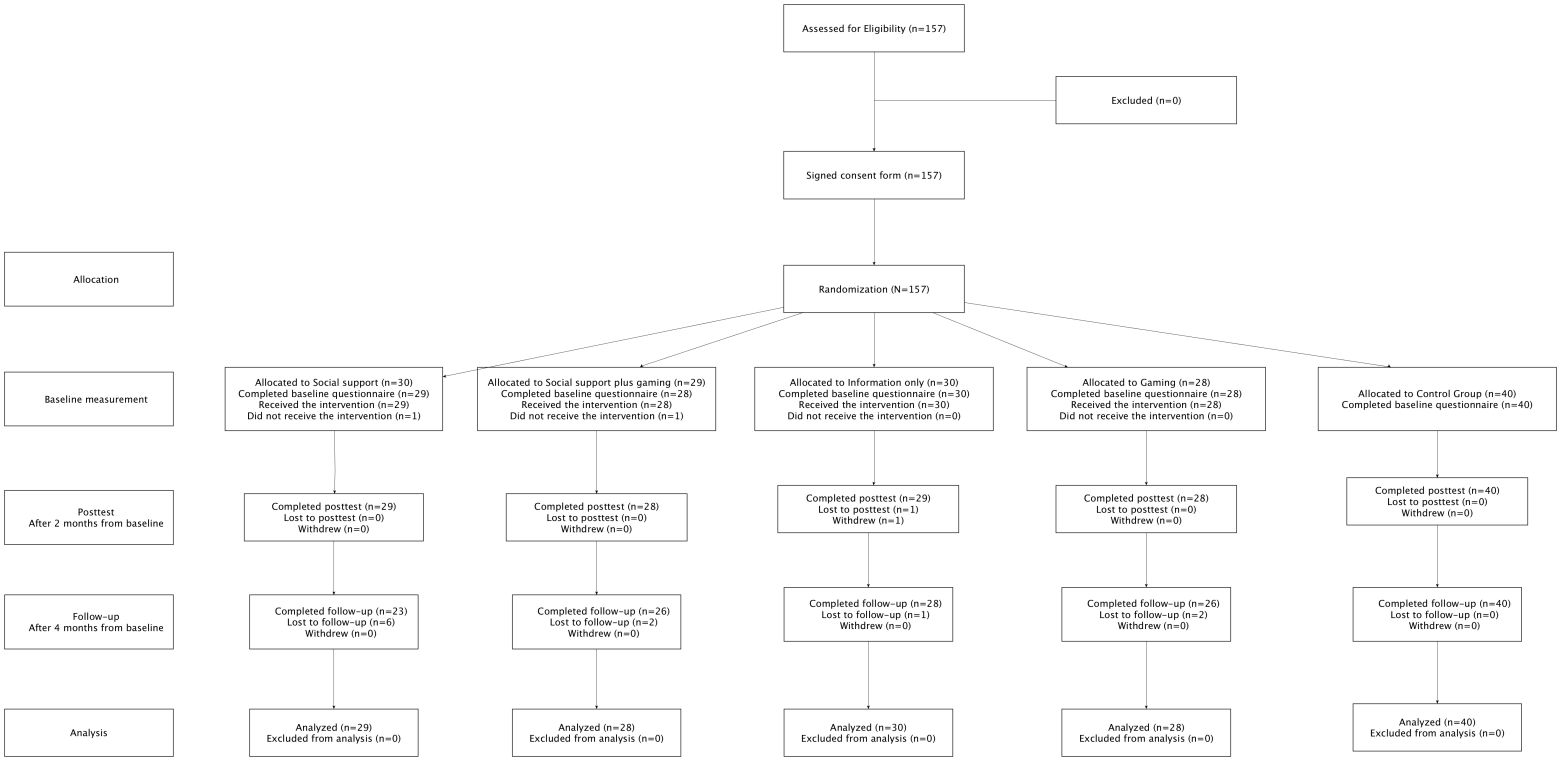
CONSORT flow diagram of participants.

Figure 4 shows the flow diagram summarizing the recruitment, randomization, attrition, and measurements at the 3 time points [57].

When the experiment was finished, participating patients were invited to a press conference [58] held at the university where the researchers presented the preliminary results of the study and the real research goals of the experiment in the presence of collaborating health professionals and the local media reporters. Moreover, all participating patients were contacted (via email or phone) and were informed that the website was publicly accessible.

### Measures

#### Overview

The baseline questionnaire, the posttest, and the follow-up used the same questions and exact wording. The study used the following measures.

#### Primary Outcomes

1. Physical activity: 6 items based on the Exercise Behaviors Scale [59]. The items measured the time spent on physical activity in the last week on a scale from 0=never to 5=more than 3 hours per week. More specifically, it measured for the entire week the time spent on each of the following exercise categories: (1) stretching or strengthening exercises, (2) walk for exercise, (3) swimming or aquatic exercise, (4) bicycling, and (5) other aerobic exercise equipment (eg, rowing, skiing) and other specified aerobic exercise. The scoring of each item as reported by the original scale estimated the number of minutes spent on exercise from 0 to 180 minutes. The average sum of the items represented the mean minutes spent on exercise by each patient. The test-retest reliability of the measure in the original reference was .72
2. Health care utilization: 5 items based on Health Care Utilization Scale [60]. They were used to measure the self-reported outpatient visits to physicians, emergency room visits, nights in the hospital, chiropractic visits, and physical therapy visits. The mean number of visits indicated the health care utilization measure. The test-retest reliability of the measure in the original reference ranged from .76 to .97 for each of the 5 items.
3. Prescription medication overuse: 6 items based on the Prescription Opioid Misuse Index [61]. Each item was a yes/no question. Items were scored 1=yes and 0=no, and the sum of the items’ score was mapped to a final score ranging from 0 to 100, where the higher the score (ie, 100=full score), the more medication overuse is indicated. The items asked about RA medications and primarily pain medications. By using the Kuder-Richardson 20 coefficient (KR-20) for dichotomous variables [62], the internal consistency of the scale measured at each assessment point range from .66 to .69.

#### Secondary Outcomes

1. Rheumatoid arthritis knowledge: 15 multiple-choice questions based on the Patient Knowledge Questionnaire in RA [63]. The total sum score of the 15 items was the final measure. The test-retest reliability of the measure in the original reference was *r*=.81 and the internal consistency measured by KR-20 was .72
2. Empowerment: 12 items adapted for RA, based on the empowerment scale proposed by Spreitzer [64]. It included 4 subdimensions: meaning, competence, self-determination, and impact. Each item in the scale was measured on a 7-point Likert scale from 1=very strongly disagree to 7=very strongly agree. The internal consistency of the scale measured using Cronbach alpha at each assessment point had values ranging from .95 to .96.

#### Predictors

The predictor variables were time of measurement and experimental condition, and additionally for the models of the primary outcomes the secondary outcomes (knowledge, empowerment). Time of measurement was coded 0=baseline, 2=posttest after 2 months, and 4=follow-up after 4 months from baseline. Experimental condition was coded as 4 dummy variables for the 4 experimental groups with the control group with no access to the site as reference. Therefore, all analyses of experimental condition were in comparison to the control group.

#### Control Variables

For sociodemographic information, patients’ age (in years), gender (1=male, 2=female), coded level of education (1=elementary school, 2=middle school, 3=high school, 4=university), working status (1=yes, 2=no), and nationality (1=Swiss, 2=other) were measured. In addition, patients indicated (in years) the duration of their arthritis disease from diagnosis and the time from the first symptoms to first diagnosis. The demographic measures were included only in the baseline questionnaire.

### Data Analysis and Model Building

#### Overview

Data were analyzed using a multilevel linear modeling technique in which the 3 measurement occasions were on the first level and the patients on the second level. Multilevel modeling was chosen because it is well suited for the analysis of repeated measurements allowing for correct inferences because standard errors of the estimated model parameters will not be underestimated and consequently reduces the risk of inflation of type I errors. It is known to be robust for unbalanced and missing data [65-67]. This approach is known as growth curve modeling; it studies growth trajectories over time, examining the pattern of change and taking into account the correlation between the measurement occasions for each patient by considering random effects of the parameters and additionally estimating the error variance-covariance matrix of the repeated measurements.

The approach models the within-patients change/growth trajectory in the outcome measure by presenting 2 kinds of submodels, both consisting of linear regressions. A level 1 submodel was computed for each participant based on 3 cases; namely, the 3 measurement points. The outcome measure was regressed on time and any potential time-variant covariate (empowerment and knowledge in our case). Because there were 3 primary outcomes and 155 participants, the total number of computed regressions was 465. The level 1 model parameters defined the shape of the individual’s growth curve or change in the outcome measure over time. The intercept indicates an individual’s initial value on the outcome variable. The slope coefficient of the time variable indicates the rate of change in the primary outcome net of the secondary outcomes, and the slope coefficients of the secondary outcome variables indicate their contribution to the primary outcome net of time.

For the level 2 submodels, the parameters from level 1 were each made dependent variables in new regressions based on the 155 participants as cases. Each parameter of the level 1 submodel was regressed on the experimental condition and the sociodemographic control variables to analyze between-patient differences in the change trajectories of the measured outcome and their effect on this change. The combined submodels (levels 1 and 2) formed the final composite model that represented the multilevel model used and evaluated for each primary and secondary outcome in this study. The approach followed a previously explained modeling procedure [68,69]. We conducted an intention-to-treat analysis that considered all patients (N=155) who filled in the baseline questionnaire and it assumed missing data were missing at random. Recent research [70] showed that for all types of missing data (missing completely at random, missing at random, and missing not at random), multiple imputation is not necessary before performing longitudinal mixed model analysis.

#### Primary Outcomes

The starting point was the unconditional means model with no predictors at any level to evaluate the intraclass correlation coefficient (ICC), which identifies the share of variance between patients among the total variance and validates the relevance of using multilevel analysis. The shares were 77.3%, 72.8%, and 61.0% for the 3 outcome measures (physical activity, health care utilization, medication overuse), confirming that multilevel modeling was an appropriate method of data analysis.

Then we constructed a series of composite “full-controlled” multilevel models. At level 1, various combinations of time and time-variant explanatory variables were used; the level 2 submodels always included the experimental conditions and sociodemographic variables. Therefore, the number of full-controlled multilevel models depended on the number of combinations that could be formed from choosing level 1 explanatory variables. Given that we had 2 explanatory variables (empowerment, knowledge) in addition to time, 4 combinations were possible: a multilevel model that included only time, or time and empowerment, or time and knowledge, or all 3 variables together. A likelihood ratio test was used to assess the multilevel model fit (the composite model of both level 1 and level 2 submodels) and track the best-fit model. In addition, Akaike information criterion (AIC) was used for comparing nonnested models. The models were full controlled (ie, adjusted) because they included all measured sociodemographic variables. This was done to investigate the net effect of the experimental condition on the outcome measure in a situation in which some of the groups, in spite of randomization, differed from others in the sociodemographic variables (ie, gender, age, and education as described subsequently). After investigating different model specifications (fixed and random effects and error variance-covariance matrix structure, such as unstructured, autoregressive order 1, etc), the final reported best-fit models in addition to the fixed effects, they included a random intercept and a first-order autoregressive structure with heterogeneous variance error variance-covariance matrix. Maximum likelihood estimation was used for estimating model parameters in which the time-variant predictors and covariates were centered at their grand mean.

For making sense of the estimated parameters, the intercept indicated the average initial level of the dependent variable in the control group. Regression parameters for the experimental condition indicated differences between the groups and the control group; most importantly, the coefficients for the interaction of condition by time signaled any condition-specific trajectories, which were the major focus of this study. Parameters for the interaction of condition by secondary outcome indicated condition-specific influences of these secondary outcomes on the primary ones.

#### Secondary Outcomes

In a similar approach, we looked at the change in the secondary outcomes by regressing them on time for each patient (level 1 submodels). All sociodemographic plus experimental group variables were used again in the level 2 submodels, as done with the primary outcomes. The goal, also similar, was to investigate the effect of the experimental conditions on both empowerment and knowledge by studying the change over time of each of the latter variables and explore whether there were systematic differences between the experimental conditions and the control group after controlling for all sociodemographic variables.

### Website Usage

For testing the effect of gamification, the usage of the website was indicated by the sum of the logged visits to each of the sections 1-6 of ONESELF, which were registered in the access log table of the website’s database. When a section was entered, a new visit was counted. To look at the effect of gamification on usage, we grouped patients who had access to gaming (social support plus gaming and gaming groups) and those who did not (social support and information groups). For comparison, *t* tests and Mann-Whitney *U* tests were employed.

The models’ estimated parameters for the primary and secondary outcomes and the test statistics for website usage were considered significant at *P*≤.05. All analyses were conducted using SPSS 20 (IBM Corporation, Armonk, NY, USA).

## Results

### Patients’ Baseline Demographics

A total of 155 people participated (information: n=30; social support: n=29; gaming: n=28; social support plus gaming: n=28; control: n=40). In all, 54.2% (84/155) were males and 45.8% (71/155) females. The mean age was 57.95 (SD 12.29) years; 85.2% (132/155) were Swiss nationals and 14.8% (23/155) were another nationality. Mean time since first diagnosis was 11.89 (SD 11.47) years. For level of education, 7.1% (11/155) had elementary school, 16.8% (26/155) had middle school, 67.7% (105/155) had high school, and 8.4% (13/155) had a university degree; 43.2% (67/155) of the patients were working. Baseline information about the sociodemographic measures for each group are reported in Table 2. Multimedia Appendix 3 reports descriptive statistics about the primary and secondary outcomes for each experimental group at every assessment point.

**Table 2:** Descriptive characteristics of the 5 experimental groups (N=155).

Self-reported measures		Information (n=30)	Social support (n=29)	Gaming (n=28)	Social support plus gaming (n=28)	Control (n=40)
Age (years), mean (SD)		55.10 (10.48)	53.17 (13.29)	54.50 (12.01)	53.46 (9.96)	69.33 (6.35)
Gender (male), n (%)		17 (57)	26 (90)	24 (86)	15 (54)	2 (5)
**Education level, n (%)**						
	Elementary school	2 (7)	1 (4)	1 (4)	0 (0)	7 (18)
	Middle school	2 (7)	6 (21)	5 (18)	2 (7)	11 (28)
	High school	24 (83)	18 (64)	18 (64)	23 (82)	19 (49)
	University	1 (3)	3 (11)	4 (14)	3 (11)	2 (5)
Work status (working), n (%)		17 (57)	19 (66)	13 (46)	14 (50)	4 (10)
**Nationality, n (%)**						
	Swiss	21 (70)	24 (83)	26 (93)	27 (96)	34 (85)
	Other	9 (30)	5 (17)	2 (7)	1 (4)	6 (15)
Duration of RA from first diagnosis (years), mean (SD)		9.90 (9.34)	10.52 (9.83)	8.89 (8.22)	10.43 (8.42)	18.11 (15.93)

### Website Usage

On average, participants paid a mean 53.68 (SD 93.07) visits to the various sections of ONESELF during the period of data collection. Participants offered the gaming experience (social support plus gaming and gaming groups) visited the website a mean 66.81 (SD 112.44) times, whereas participants not offered the gaming experience visited the site less frequently (mean 26.15, SD 27.11 visits). As a result, there was a significant difference between the groups (*t*_91_=–2.41, *P*=.02; *U*=812, *P*=.02). This suggests that groups who were offered the gamified experience used the website more often than groups denied this experience.

### Multilevel Model Evaluation

#### Primary Outcomes

For the best-fit multilevel models, the estimated fixed effects parameters (the interactions between time-variant covariates and experimental groups) are reported for the 3 outcome measures in Table 3. For a better overview, only the significant parameters are shown. The complete regression tables, including *P* values, standard errors, etc, are available in Multimedia Appendix 4.

Physical activity was best predicted by a model that included time and empowerment. Looking at the trajectory of the control group, initially the mean number of minutes spent on exercise at baseline was significant (unstandardized beta coefficient [B]=57.55, *P*<.001) and there were no differences at baseline between the experimental groups and the control group. There was a significant influence factor: mean minutes spent on exercise increased over time for patients with access to the social support sections and gaming (B=3.39, *P*=.02), indicating that patients with access to the complete website features (social support plus gaming group) became more physically active as the experiment progressed in comparison to the group with no access to the site.

Change in health care utilization was best predicted by a model that included time and empowerment. At baseline, there was, on average, significant utilization of the health care system (B=2.79, *P*=.02) and there were no differences between the experimental conditions and the control group. As time passed, the average rate of change in health care utilization showed a significant decrease for patients in the social support group (B=–0.41, *P*=.01) and patients in the social support plus gaming group (B=–0.33, *P*=.03).

Prescription medication overuse was best predicted by a model that included time and empowerment. At baseline, the control group showed a significant medication overuse (B=12.06, *P*=.03), with no differences found between the control group and patients in the social support group or the gaming group. However, there were differences in the initial status between the control group and patients in the social support plus gaming group (B=9.51, *P*=.03) and patients in the information-only group (B=10.06, *P*=.02). When considering time, the mean monthly rate of change in medication overuse showed a decrease (marginally significant) only for patients in the social support group (B=–1.61, *P*=.056).

**Table 3:** Estimates of regression coefficients for predicting change in primary outcomes.

Predictors		Primary outcomes,^a^ B		
		Physical activity	Health care utilization	Medication overuse
Intercept		57.55^b^	2.79^c^	12.06^c^
**Group**				
	Social support	NS	NS	NS
	Social support plus gaming	NS	NS	9.51^c^
	Information	NS	NS	10.06^c^
	Gaming	NS	NS	NS
**Time**		NS	NS	NS
	Social support × time	NS	–0.41^c^	–1.61^c^
	Social support plus gaming × time	3.39^c^	–0.33^c^	NS
	Information × time	NS	NS	NS
	Gaming × time	NS	NS	NS
**Empowerment**		NS	NS	NS
	Social support × empowerment	NS	NS	NS
	Social support plus gaming × empowerment	NS	NS	NS
	Information × empowerment	NS	NS	NS
	Gaming × empowerment	NS	NS	NS

^a^ NS: not significant

^b^ *P*<.001

^c^ *P*≤.05

#### Secondary Outcomes

A significant change in empowerment without differences between the experimental conditions in the initial status is visible in the significant intercept (B=51.56, *P*<.001) and the absence of significant parameters for the experimental groups (Table 4). Generally, the mean rate of change for the control group did not increase over time as indicated by an insignificant parameter of the time variable. However, the interaction of 2 of the experimental conditions and time indicates that for these 2 groups the increase in empowerment became larger over time (B=2.59, *P*=.03; B=2.29, *P*=.05; respectively). The 2 groups were the social support and gaming groups that had access also to the informative sections of course. This means that users of the website with access to either social support sections or the gaming experience, both in addition to the informative parts, gained more empowerment than patients without access to the website at all.

The control group initially had a significant level of RA knowledge (B=5.89, *P*<.001) because there were no differences in the initial status between the experimental and the control group. Moreover, the effect of time was not significant where all the experimental conditions were comparable and had an average rate of change similar to the control group (equal to zero; null hypothesis was retained). This indicated that knowledge did not change significantly during the entire intervention for any of the experimental conditions.

**Table 4:** Estimates of regression coefficients for predicting change in secondary outcomes.

Predictors		Secondary outcome,^a^ B	
		Empowerment	RA knowledge
Intercept		51.56^b^	5.89^b^
**Group**			
	Social support	NS	NS
	Social support plus gaming	NS	NS
	Information	NS	NS
	Gaming	NS	NS
**Time**		NS	NS
	Social support × time	2.59^c^	NS
	Social support plus gaming × time	NS	NS
	Information × time	NS	NS
	Gaming × time	2.29^c^	NS

^a^ NS: not significant

^b^ *P*<.001

^c^ *P*≤.05

## Discussion

### Principal Findings

The study included cognitive and behavioral measures. It tested the effect of online social support and gamification on health outcomes (health care utilization and medication overuse), patients’ behavior (physical activity), empowerment, and knowledge for patients with RA.

Empowerment levels changed over time, more so in groups having access to online social support or a gamified experience of the website. The study suggests that online social support plays an essential role in improving patients’ level of empowerment. This observation contrasts with the results of the earlier study done by Camerini and Schulz on ONESELF [55], which did not find any effect for functional interactivity on empowerment. This might be because we considered all 4 dimensions of empowerment together, whereas the previous study treated each dimension as a single variable. Moreover, our study included gamification, which was not included in Camerini and Schulz [55], in which functional interactivity was primarily about access to social support features (forum and chat room). Lastly, this study had 3 assessment points targeting RA patients, whereas the previous study was a pre-post test design targeting fibromyalgia syndrome patients. In addition, our comparisons were always with a control group without access to the website; the previous study did not have a real control group [55].

In contrast, there were no changes of RA knowledge levels over time for any of the experimental conditions. This result shares the findings of Camerini and Schulz [55] in which functional interactivity did not have a direct impact on knowledge of patients. This was additionally observed in this study by the absence of RA knowledge as a predictor in any of the best-fit models. The fact that knowledge did not change during the entire intervention for any of the experimental groups would explain the absence of its effect on the primary outcomes and not being part in any best-fit model, in contrast to empowerment. Given the familiarity of the participants in this study with their RA condition (mean time since first diagnosis was 11.89 years), their knowledge was sufficient to a certain extent and one would not expect significant change. The effect of knowledge might be visible if participants were much younger and recently diagnosed with RA. It would be an interesting idea for further research to test similar experiments with much earlier diagnosed patients.

Gamified experience of the website showed to increase website usage as defined by the number of access times to meaningful website sections. Because the main goal of gamifying systems is to engage users and increase their participation [40,48,71], a gamified health website such as ours also attracts and encourages patients to use and interact with the platform more. This adds empirical evidence to the validity of the motives behind gamification in the medical field. Moreover, gamification as implemented and designed for this experiment showed to increase empowerment over time. Patients’ participation in a competition-like environment where their actions, contributions, and usage of the website were rewarded might have increased their motivation and confidence in acquiring and processing the disease-related information they found on ONESELF, eventually improving or creating the sense of empowerment in dealing with RA. Because empowerment and its importance were celebrated throughout the literature [10], having an association between gamification and empowerment provides additional evidence of its benefits. Further experiments are needed to better understand the mechanisms and the relation in greater detail of how presenting health information in an environment using gaming elements would eventually affect empowerment levels in managing a chronic disease.

Gamification and online social support was also associated with increased physical exercise over time. Looking to this effect from the gaming part, the increased physical activity aligns with a similar observation reported by Hamari et al [43] for a gamified platform in the context of fitness and exercise websites. From the online social support part, the increase in patients’ self-reported physical activity echoes the effect that tested an Internet-based physical activity program with individually tailored supervision, exercise equipment, and group contacts compared to a control group that had access to Internet-based program offering general information on exercises and physical activity [20]. The combined effect of online social support and the gaming experience suggests that advice and information received by patients in the social support plus gaming group helped them cope with their disease and promoted exercises suitable for them, allowing the patients to follow and adhere more to the information and advice especially when they were presented in a gaming context. That is also evident in the case of health care utilization where a significant decrease was associated with gamification.

In the same line, this was also true for patients having access to online social support. The contribution of the information exchanged through the forum and chat room, which were the major features in the social support group, seems to improve patients’ ability to self-manage their condition and eventually reduce the need to use the physical health care system. In other words, sharing the experiences and raising questions with other RA patients and health care professionals online substituted for the need to overly use the real health care system. Something similar happened in the case of medication overuse, in which patients accessing online social support features witnessed a decrease (marginally significant) compared to the control group. This complements and adds to the experiment done by Lorig et al [9] that looked into the effects of delivering an Internet-based arthritis self-management program compared to a usual care, small-group, self-management program by providing evidence of the role of Web-based delivered programs in decreasing health care utilization and medication overuse for RA patients.

For this study, we cannot conclude which is better: online social support or gamification. In fact, the relation between both components should not be represented as an exclusive OR as much as a complimentary relation. As shown in the growth curve models for each measured outcome except knowledge, patients with access to either of them (social support or gaming) or both of them (social support plus gaming) had an improvement leading to a better health or behavioral outcome.

### Limitations

The study, as many others on Web-based interventions, used self-reported measures to assess changes in behavioral and health outcomes. More objective measures could be utilized to study the efficacy of gamification and social support that would complement the results presented. This is especially pertinent for the case of measuring physical activity, for which other studies did not find differences when physical equipment (ie, activity monitor) was used to measure the participants’ physical exercise or activity [20,71].

Moreover, a 4-month period might not be sufficient to explore the longitudinal effect of the experimental condition on the changes in measured outcomes. A longer period with more measurement points would result in more precise estimates of the effect of the experimental manipulation on the growth curves that might take different functional forms than the linear one used in this study.

Although they did not have access to ONESELF, we cannot guarantee that the control group in the current study did not use other online tools and platforms on their own that provide information or any other support for patients with RA.

In addition, the study did not include a formal measure for computer skills or the ability and confidence to navigate and browse the Internet. It was only self-reported by patients and it would be interesting to include a formal measure (ie, e-literacy scale) in further studies to understand and identify patterns of intervention use among patients with different levels of computer skills.

Finally, another important aspect to keep in mind is the context in which the study was conducted. It was in the Italian-speaking part of Switzerland (Canton Ticino). However, we do not see this as a limitation as much as a call for more research in this area in different sociocultural contexts to be able to generalize the current study findings.

### Conclusions

The Web-based intervention had a positive impact (more desirable outcomes) on intervention groups compared to the control group. Social support sections on the website decreased health care utilization and medication overuse and increased empowerment. Gamification alone or with social support increased physical activity and empowerment and decreased health care utilization. The information-only condition did not differ from the control group. For the other measures, all groups were comparable to the control group.

In conclusion, this study pointed to the positive potential and the promising desirable effects of online social support and especially gamification on patients’ behavioral and health outcomes when included in an eHealth intervention. Hence, more research on the efficacy of gamification applied in different situations (ie, patients diagnosed with different diseases, different targeted platforms such as mobile apps) is needed to understand which gamification strategies would help the most in benefiting the patients and meeting the objectives and goals of health platforms. Moreover, the concept of patient empowerment as operationalized and measured in this study showed to be an important construct in the best-fit models predicting and representing the change in patients’ health and behavioral outcomes. In addition, the study showed that patients who had access only to informational webpages were similar to the control group who had no access to the website. This might suggest that eHealth Web-based interventions would benefit from including social support features or gamification in their platforms compared to informative sections alone.

Lastly, the choice of gamifying a website (as in this study) and incorporating game design elements and corresponding mechanisms instead of designing a new game from scratch has high practical implications. Because many health-related websites exist and contain already a substantial amount of health information, they can rapidly benefit from the gamification done in this study. By adjusting their Web-based platforms and redefining their users’ experience on the website through the inclusion of gaming elements (ie, badges, points, rewards, incentives) and presenting challenges and competition or an action-reward environment, owners of health websites will be able to create a new type of interaction between online users and the already existing health information found on their websites.

## Multimedia Appendix 1

Video Demos that demonstrate various aspects of the RCT (various sections and features of the ONESELF intervention, types of access by experimental groups and the implemented gamification mechanism).

## Multimedia Appendix 2

The brochure and the consent form that were distributed to the patients.

## Multimedia Appendix 3

Descriptive statistics about the primary and secondary outcomes for each experimental group in every assessment point.

## Multimedia Appendix 4

Primary and secondary outcomes multilevel models (fixed and random-effect estimated parameters, error variance-covariance matrix, standard errors, p-values and 95% confidence intervals in addition to model fit measures).

## Multimedia Appendix 5

CONSORT-EHEALTH checklist V1.6.2 [57].

## Abbreviations

AIC: Akaike information criteria
CMS: content management system
HON: Health on the Net
ICC: intraclass correlation coefficient
ISG: Internet support group
NS: not significant
RA: rheumatoid arthritis
RCT: randomized controlled trial
