# Supplementary material for: The Effect of Social Support Features and Gamification on a Web-Based Intervention for Rheumatoid Arthritis Patients: Randomized Controlled Trial
Source: J Med Internet Res. 2015 Jan 9;17(1):e14. doi: 10.2196/jmir.3510 (PMC4296094; doi:10.2196/jmir.3510)
Supplement: Supplementary file 2 [file jmir_v17i1e14_app2.pdf]

Nicht frankieren
Ne pas affranchir
Non affrancare

A

Geschäftsantwortsendung Invio commerciale risposta
Envoi commercial-réponse

Università della Svizzera italiana
Institute of Communication and Health (ICH)
Zlatina Kostova
Via Giuseppe Buffi 13
6900 Lugano

Ritagliare lungo le parti tratteggiate e rispedire la cartolina risposta preaffrancata

Chi contattare?

Siamo alla vostra completa disposizione per qualsiasi informazione o chiarimento:

Zlatina Kostova
Tel. +41 (0)58 666 45 89
Email: zlatina.kostova@usi.ch

Ahmad Allam
Tel. +41 (0)58 666 45 89
Email: oneself.switzerland@gmail.com

Teresa Cafaro
Tel. +41 (0)58 666 44 87
Email: oneself.switzerland@gmail.com

Università della Svizzera italiana

Facoltà di scienze della comunicazione

Institute of Communication and Health ICH

Lega ticinese per la lotta contro il reumatismo
Il movimento è salute

Soffrite di Artrite o di altre malattie reumatiche?

Vi invitiamo a consultare il sito
www.oneself.ch

ONESELF

TUTTO SULLE MALATTIE REUMATICHE

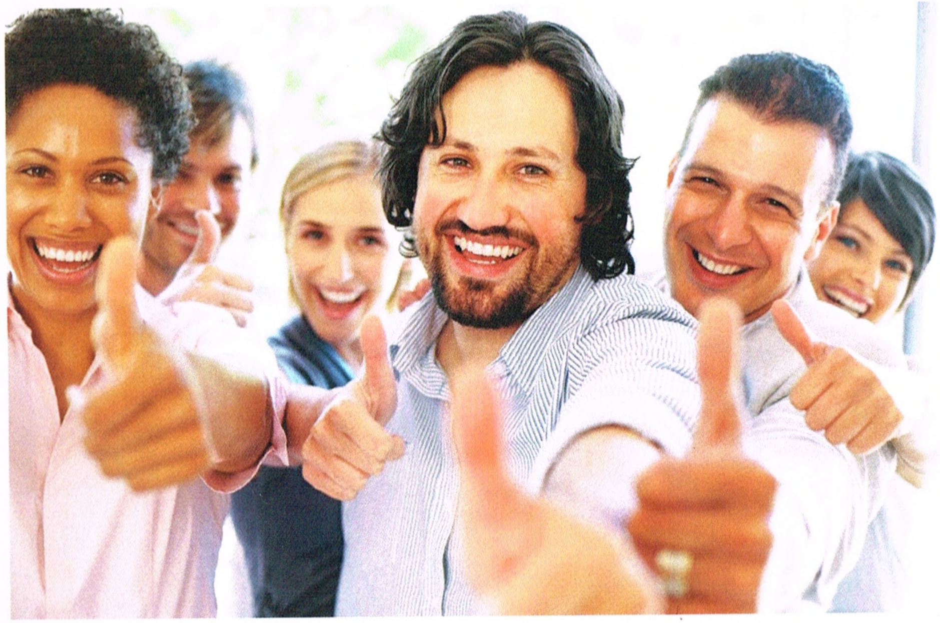

Gentile Signora, Egregio Signore,

Grazie per aver accettato di partecipare allo studio sull'artrite.

Scopo dello studio

L'università della Svizzera italiana in collaborazione con la Lega Ticinese contro il Reumatismo e il Fondo Nazionale Svizzero conduce uno studio sull'importanza del sostegno sociale per le persone che soffrono di artrite o di altre malattie reumatiche. Lo studio ha come obiettivo di fornire ai pazienti il supporto e le informazioni necessarie per aiutarli ad affrontare le difficoltà legate a queste condizioni croniche. Abbiamo creato un sito Internet dedicato a voi - le persone che soffrono di problemi reumatici, dandovi la possibilità di condividere le vostre esperienze, ricevendo consigli dai medici specialisti, tanti suggerimenti pratici e informazioni per come gestire meglio questi disturbi cronici.

Quale sarà il Suo impegno? In realtà molto facile e veloce...

1. La invitiamo prima a registrarsi sul sito www.oneself.ch collegandosi alla sezione **artirite** specificando alcune informazioni che rimarranno anonime e scegliendo un username e una password. È gentilmente pregato/a di indicare, nel momento della registrazione, il suo nome e un recapito telefonico.
2. Una volta registrato/a le chiediamo gentilmente di compilare un questionario che troverà online. La compilazione del questionario richiederà circa 15 minuti. Un simile questionario dovrà essere compilato altre 2 volte dopo la fine dello studio per valutare l'eventuale effetto del sito.
3. Dopo aver compilato la prima volta il questionario le chiederemo di "interagire" con il sito per un periodo di circa 5-6 settimane per un'oretta alla settimana.
4. I ricercatori che conducono la ricerca e che gestiscono il sito la contatteranno per invitarla a collegarsi al sito settimanalmente e saranno a sua completa disposizione per qualsiasi chiarimento in merito.

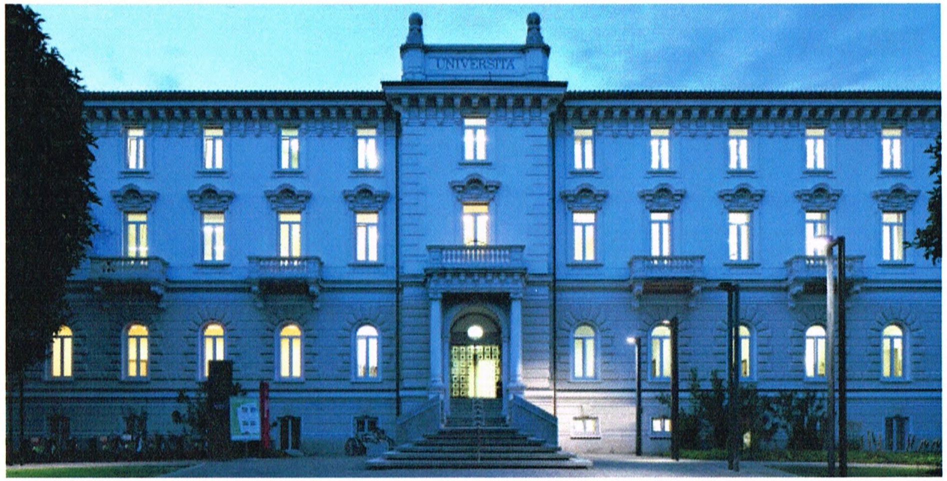

Dichiarazione di consenso

Attesto che mi è stata consegnata l'informazione scritta per i pazienti, ne ho preso visione e ne ho compreso il contenuto. In caso di incertezze future potrò richiedere in ogni momento ulteriori informazioni al mio medico curante o ai coordinatori del sito.

I miei dati verranno conservati con la garanzia della protezione secondo la legge che la regola. Ho la possibilità in ogni momento e senza motivare la mia decisione di interrompere la collaborazione, senza che ciò mi comporti alcun svantaggio per il trattamento della mia malattia.

Consenso scritto del/la paziente

Luogo e data ______________________________

Nome e cognome del/la paziente ______________________________

Telefono: ______________________________

Firma ______________________________

Ritagliare lungo le parti tratteggiate e rispedire la cartolina risposta preaffrancata

Quale è il Suo vantaggio personale?

- La sua partecipazione a un sito realizzato da ricercatori universitari e medici reumatologi le darà informazioni rilevanti e utili per la gestione della sua malattia, avendo un supporto altamente professionale e specializzato.
- La sua collaborazione contribuirà a fornire preziose conoscenze per migliorare il supporto dei pazienti che soffrono di malattie reumatiche e aiuterà a tante altre persone nella sua stessa condizione.

Considerazioni di ordine etico

La ricerca ha intenti puramente scientifici e per questo motivo la sua opinione è molto importante per noi. Lo studio ha ricevuto l'approvazione da parte del Comitato etico cantonale. La sua partecipazione allo studio è volontaria e lei potrà revocare il suo consenso alla partecipazione in qualsiasi momento senza fornire alcuna ragione e senza conseguenze.

Università della Svizzera italiana

Facoltà di scienze della comunicazione

Institute of Communication and Health ICH

Lega ticinese per la lotta contro il reumatismo
Il movimento è salute

ONESELF è un progetto pronosso dall'**Institute of Communication and Health** dell'USI Università della Svizzera italiana, realizzato con la collaborazoine e il sostegno della **Lega Ticinese per la Lotta Contro il Reumatismo** e con il **Fondo Nazionale Svizzero**.

Per qualsiasi informazione o suggerimento siamo a vostra disposizione!

Contattate:

Zlatina Kostova
USI Università della Svizzera italiana
Institute of Communication and Health
Via Giuseppe Buffi 13
6900 Lugano
tel: +41 58 666 4621
oneself.switzerland@gmail.com

www.ich.com.usi.ch

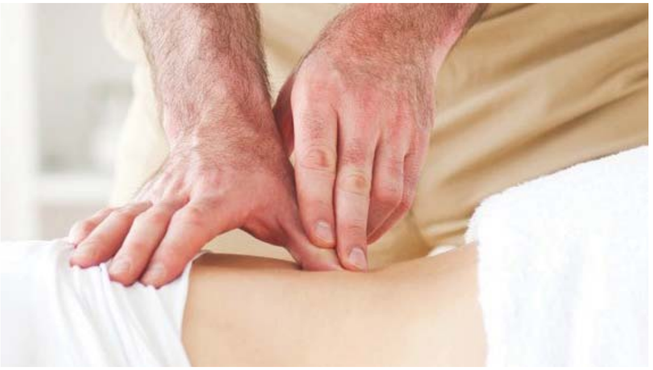

"Se io appena appena posso, devo reagire!
Questo è il consiglio che io do ad altra gente con questa malattia: non vi abbattete! Nella vita bisogna provare e tentare tutte le strade possibili e immaginabili, mai fermarsi.
Questo è il grande insegnamento che l'artrite mi ha dato.
La vita ce la dobbiamo godere tutta, giorno dopo giorno"

(Rosa, 49 anni, lotta contro l'artrite da 10 anni)

FNSNF
FONDO NAZIONALE SVIZZERO
PER LA RICERCA SCIENTIFICA

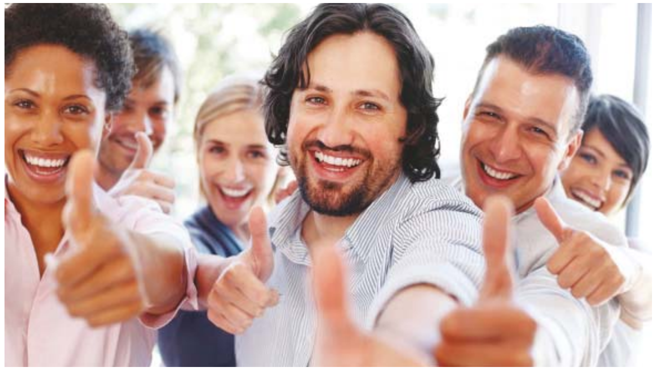

Soffrite di Artire?

Vi invitiamo a consultare il sito
www.oneself.ch

ONESELF
TUTTO SULLE MALATTIE REUMATICHE

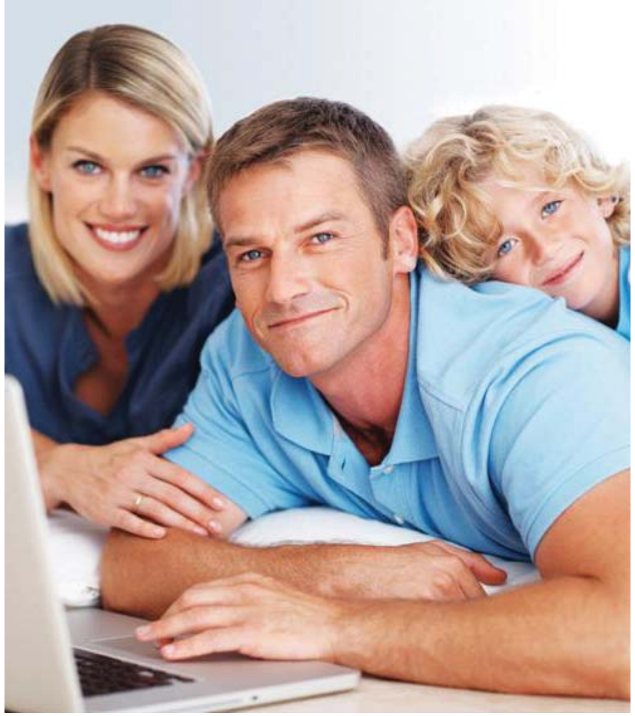

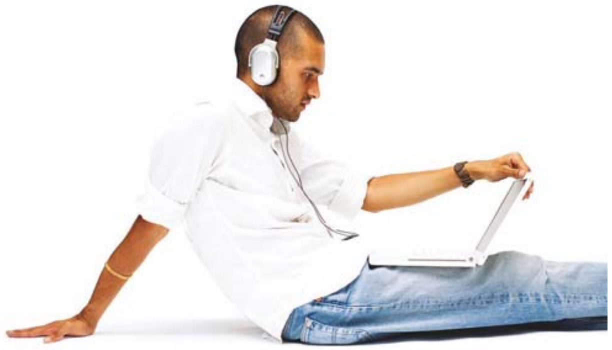
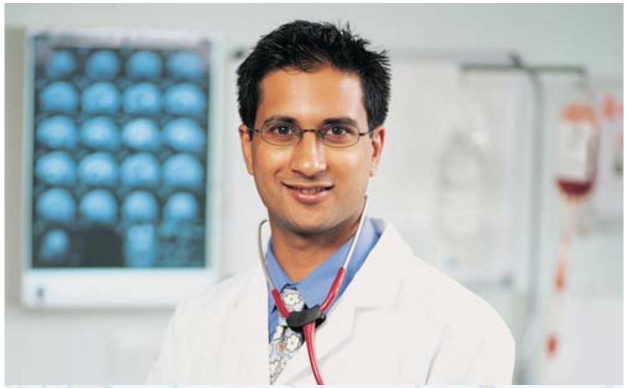
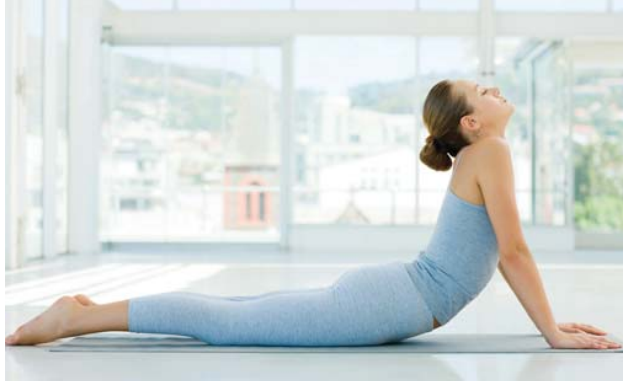
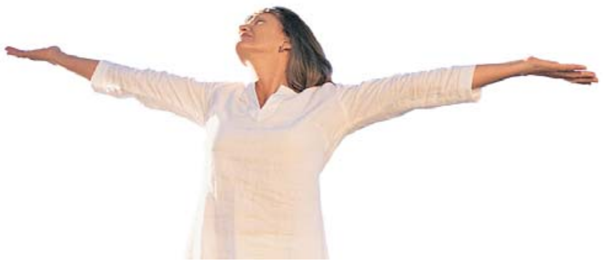

Che cos'è Oneself?

La battaglia contro l'artrite reumatoide (AR) non è certo una sfida facile: lo shock per un corpo che ci ha improvvisamente traditi, le difficoltà, le preoccupazioni, le limitazioni, la rabbia, la frustrazione possono farci anche più male delle nostre articolazioni.

Ma vincere la nostra battaglia è possibile: grazie ai nuovi farmaci, alle terapie e alla forza di volontà, se non possiamo eliminare l'artrite, possiamo bloccarla, possiamo impedirle di condizionarci, possiamo riprenderci e goderci la nostra vita. Per farlo, dobbiamo curarci, dobbiamo lottare e non dobbiamo farlo da soli. Per questo il team di specialisti di **ONESELF** ha creato, in collaborazione con la Lega ticinese contro il reumatismo e l'Università della Svizzera italiana, un sito dove potremo tutti condividere le nostre esperienze e sostenerci a vicenda, chiedere consigli agli specialisti, avere informazioni chiare e suggerimenti su come convivere al meglio con la malattia mantenendo una buona qualità di vita e aprendoci a nuove prospettive.

Tutti i materiali sono stati creati con medici, terapeuti e psicologi professionisti nell'ambito delle malattie reumatiche e con ricercatori universitari, allo scopo di aiutarci e nella speranza che, come scriveva un poeta tanti anni fa, nonostante tutto, la nostra anima resti invincibile.

Novità

Ecco cosa potete trovare su **ONESELF** all'indirizzo internet:
www.oneself.ch

Fermare l'artrite
Le terapie con i nuovi farmaci, la fisioterapia, l'ergoterapia e il contributo che ognuno può dare nella lotta contro l'AR. I testi sono accompagnati da immagini e video per illustrare al meglio tutte le informazioni.

Vivere con l'artrite
Suggerimenti su come affrontare l'impatto dell'AR sulla quotidianità, senza lasciarsi condizionare e mantenendo una buona qualità di vita

Per saperne di più
Informazioni chiare sull'artrite, su come riconoscerla, sulle sue cause e su che cosa succede alle nostre articolazioni

Lo specialista risponde
Lo spazio dove fare tutte le domande che si vuole a medici, fisioterapisti e terapeuti

Per i famigliari
La malattia ha un impatto anche sui nostri famigliari, che insieme a noi devono accettare la malattia.
Qui alcuni consigli per aiutarli.
A presto su www.oneself.ch
