# Supplementary material for: The Effect of Social Support Features and Gamification on a Web-Based Intervention for Rheumatoid Arthritis Patients: Randomized Controlled Trial
Source: J Med Internet Res. 2015 Jan 9;17(1):e14. doi: 10.2196/jmir.3510 (PMC4296094; doi:10.2196/jmir.3510)
Supplement: Supplementary file 3 [file jmir_v17i1e14_app3.pdf]

Throughout this appendix, the abbreviations below will be used for representing the experimental groups.
Info: Information only group; Gaming: Gaming group; SocSup: Social support group; SocSupGaming: Social support plus gaming; CG: Control group

Descriptive statistics (Mean, SD) for primary outcomes for experimental groups at time 1 (baseline), time 2 (posttest) and time 3 (follow-up)

Time	Experimental Group		Amount of exercise	Health care utilization	Medication overuse
1	SocSup	Mean	32.41	3.69	6.90
		N	29.00	29.00	29.00
		SD	31.92	3.14	10.46
	SocSupGaming	Mean	26.96	3.50	10.71
		N	28.00	28.00	28.00
		SD	27.10	2.95	20.39
	InfO	Mean	30.67	3.23	8.33
		N	30.00	30.00	30.00
		SD	23.13	3.45	15.63
	Gaming	Mean	33.57	2.82	4.17
		N	28.00	28.00	28.00
		SD	32.59	2.54	9.76
	Control Group	Mean	50.50	1.75	4.58
		N	40.00	40.00	40.00
		SD	22.03	2.06	11.93
2	SocSup	Mean	28.10	3.00	6.32
		N	29.00	29.00	29.00
		SD	29.68	3.56	12.92
	SocSupGaming	Mean	28.21	3.50	8.33
		N	28.00	28.00	28.00
		SD	27.39	3.56	16.04
	InfO	Mean	33.36	3.38	14.37
		N	29.00	29.00	29.00
		SD	26.55	3.10	22.59
	Gaming	Mean	33.30	2.46	2.98
		N	28.00	28.00	28.00
		SD	32.43	2.28	7.93
	Control Group	Mean	44.12	1.45	2.14
		N	40.00	40.00	39.00
		SD	20.69	1.72	5.64
3	SocSup	Mean	33.59	2.35	2.90
		N	23.00	23.00	23.00
		SD	30.47	3.28	8.18
	SocSupGaming	Mean	30.38	2.46	5.77
		N	26.00	26.00	26.00
		SD	27.86	2.77	11.49
	InfO	Mean	29.37	3.14	9.52
		N	28.00	28.00	28.00

		SD	20.95	3.32	17.23
	Gaming	Mean	36.15	2.19	.00
		N	26.00	26.00	26.00
		SD	34.26	1.67	.00
	Control Group	Mean	44.13	1.50	2.92
		N	40.00	40.00	40.00
		SD	20.59	1.69	11.25

SD: Standard deviation; N: number of participants

Descriptive statistics (Mean, SD) for secondary outcomes for experimental groups at time 1 (baseline), time 2 (posttest) and time 3 (follow-up)

Time	Experimental Group		Empowerment	RA knowledge
1	SocSup	Mean	48.10	7.78
		N	29.00	29.00
		SD	13.00	2.16
	SocSupGaming	Mean	49.86	7.19
		N	28.00	28.00
		SD	16.48	1.94
	InfO	Mean	48.67	6.97
		N	30.00	30.00
		SD	17.97	2.03
	Gaming	Mean	46.86	7.61
		N	28.00	28.00
		SD	17.93	1.97
	Control Group	Mean	44.33	6.40
		N	36.00	40.00
		SD	26.87	2.21
2	SocSup	Mean	49.41	8.07
		N	29.00	29.00
		SD	11.71	1.82
	SocSupGaming	Mean	51.18	7.51
		N	28.00	28.00
		SD	16.17	2.27
	InfO	Mean	47.07	7.24
		N	29.00	29.00
		SD	16.68	2.39
	Gaming	Mean	50.68	8.22
		N	28.00	28.00
		SD	15.94	2.14
	Control Group	Mean	48.20	6.45
		N	35.00	40.00
		SD	23.56	2.15
3	SocSup	Mean	51.09	8.64
		N	23.00	23.00

		SD	14.49	1.90
	SocSupGaming	Mean	48.12	7.74
		N	26.00	26.00
		SD	16.33	2.03
	InfO	Mean	50.96	8.15
		N	28.00	28.00
		SD	16.11	2.01
	Gaming	Mean	49.54	8.63
		N	26.00	26.00
		SD	16.17	1.89
	Control Group	Mean	46.62	6.52
		N	34.00	40.00
		SD	23.61	2.04
