# Supplementary material for: The Effect of Social Support Features and Gamification on a Web-Based Intervention for Rheumatoid Arthritis Patients: Randomized Controlled Trial
Source: J Med Internet Res. 2015 Jan 9;17(1):e14. doi: 10.2196/jmir.3510 (PMC4296094; doi:10.2196/jmir.3510)
Supplement: Supplementary file 4 [file jmir_v17i1e14_app4.pdf]

Table 1: Overview of experimental conditions

Group access	No access to information sections		Access to informational sections	
	No access to social support features	Access to social support features	No access to social support features	Access to social support features
No access to gaming feature	Control Group (CG)		InfO	SocSup
Access to gaming feature			Gaming	SocSupGaming

Throughout this appendix, the abbreviations below will be used in the following reported models.

Info: Information only group; Gaming: Gaming group; SocSup: Social support group; SocSupGaming: Social support plus gaming; CG: Control group

Primary Outcomes

Table 2: Multilevel model for physical activity outcome measure (minutes spent on exercise)

Parameter	Estimate	Std. Error	Sig.	95% Confidence Interval	
				Lower Bound	Upper Bound
**Fixed effects**					
Intercept	57.552	11.312	.000	35.237	79.867
RA Duration	.238	.223	.28	-.202	.680
Age	-.103	.257	.68	-.610	.403
Male	-15.531	5.979	.01	-27.330	-3.732
Elementary school	-16.809	15.022	.26	-46.448	12.828
Middle school	-17.965	9.531	.06	-36.764	.834
High school	-18.568	8.273	.02	-34.885	-2.252
Swiss	6.269	6.691	.35	-6.935	19.473
Working	6.135	5.939	.30	-5.580	17.852
SocSup	-6.561	9.706	.50	-25.709	12.587
SocSupGaming	-14.419	8.823	.10	-31.820	2.980
InfO	-5.172	8.571	.54	-22.083	11.737
Gaming	-2.083	9.509	.82	-20.843	16.675
Time	1.138	2.043	.57	-2.900	5.178
RA Duration X Time	-.015	.038	.69	-.091	.061
Age X Time	.085	.047	.07	-.007	.179
Male X Time	.750	1.084	.49	-1.393	2.895
Elementary school X Time	-4.348	2.741	.11	-9.765	1.068
Middle school X Time	-2.642	1.734	.13	-6.068	.784
High school X Time	-2.210	1.509	.14	-5.191	.770
Swiss X Time	-.834	1.214	.49	-3.234	1.566
Working X Time	-1.725	1.092	.11	-3.883	.432
SocSup X Time	2.354	1.740	.17	-1.086	5.795
SocSupGaming X Time	3.396	1.542	.02	.346	6.446
InfO X Time	1.018	1.534	.50	-2.014	4.051
Gaming X Time	2.255	1.678	.18	-1.063	5.575

Empowerment	-.159	.437	.71	-1.019	.700
RA Duration X Empowerment	-.019	.007	.01	-.034	-.004
Age X Empowerment	-.004	.008	.58	-.022	.012
Male X Empowerment	-.079	.193	.68	-.459	.301
Elementary school X Empowerment	.193	.540	.72	-.868	1.255
Middle school X Empowerment	.647	.361	.07	-.062	1.358
High school X Empowerment	.444	.326	.17	-.197	1.085
Swiss X Empowerment	.205	.216	.34	-.220	.631
Working X Empowerment	-.153	.206	.45	-.557	.252
SocSup X Empowerment	-.382	.356	.28	-1.082	.318
SocSupGaming X Empowerment	-.231	.309	.45	-.839	.377
InfO X Empowerment	-.141	.288	.62	-.707	.425
Gaming X Empowerment	-.089	.335	.78	-.749	.570
**Random effects**					
σ^2 between participants					
Intercept	431.240	62.989	.000	323.882	574.184
σ^2 within participants (error covariance structure) Heterogeneous 1st order autoregressive					
σ_1^2	307.519	48.006	.000	226.460	417.593
σ_2^2	140.075	45.817	.002	73.780	265.938
σ_3^2	79.595	24.040	.001	44.034	143.873
ρ	.164	.163	.31	-.161	.457
**Model fit indices**					
-2LL	3585.674				
AIC	3673.674				

Table 3: Multilevel model for health care utilization outcome measure

Parameter	Estimate	Std. Error	Sig.	95% Confidence Interval	
				Lower Bound	Upper Bound
**Fixed effects**					
Intercept	2.794	1.262	0.02	0.303	5.284
RA Duration	0.008	0.025	0.74	-0.041	0.057
Age	-0.029	0.029	0.31	-0.086	0.027
Male	-0.032	0.667	0.96	-1.348	1.284
Elementary school	-1.001	1.676	0.55	-4.308	2.307

Middle school	-0.784	1.065	0.46	-2.885	1.316
High school	0.104	0.924	0.91	-1.719	1.928
Swiss	-0.064	0.746	0.93	-1.537	1.408
Working	-1.382	0.662	0.03	-2.688	-0.076
SocSup	1.676	1.082	0.12	-0.459	3.811
SocSupGaming	1.588	0.982	0.10	-0.349	3.526
InfO	1.252	0.956	0.19	-0.634	3.138
Gaming	0.511	1.060	0.63	-1.580	2.603
Time	-0.181	0.206	0.38	-0.589	0.227
RA Duration X Time	0.007	0.004	0.09	-0.001	0.014
Age X Time	0.006	0.005	0.24	-0.004	0.015
Male X Time	0.125	0.109	0.25	-0.091	0.340
Elementary school X Time	0.074	0.276	0.78	-0.472	0.621
Middle school X Time	0.023	0.175	0.89	-0.324	0.370
High school X Time	-0.015	0.153	0.92	-0.317	0.288
Swiss X Time	-0.012	0.122	0.92	-0.253	0.229
Working X Time	0.309	0.110	0.006	0.091	0.526
SocSup X Time	-0.416	0.175	0.01	-0.762	-0.070
SocSupGaming X Time	-0.332	0.155	0.03	-0.638	-0.025
InfO X Time	-0.092	0.154	0.55	-0.397	0.214
Gaming X Time	-0.176	0.168	0.29	-0.510	0.157
Empowerment	0.033	0.048	0.49	-0.062	0.128
RA Duration X Empowerment	0.001	0.001	0.26	-0.001	0.003
Age X Empowerment	0.002	0.001	0.02	0.000	0.004
Male X Empowerment	-0.010	0.021	0.65	-0.051	0.032
Elementary school X Empowerment	-0.089	0.060	0.14	-0.207	0.030
Middle school X Empowerment	-0.061	0.041	0.13	-0.141	0.019
High school X Empowerment	-0.050	0.037	0.17	-0.123	0.023
Swiss X Empowerment	0.004	0.023	0.88	-0.042	0.049
Working X Empowerment	0.016	0.022	0.47	-0.028	0.060
SocSup X Empowerment	0.041	0.039	0.29	-0.035	0.117
SocSupGaming X Empowerment	0.040	0.034	0.23	-0.026	0.107
InfO X Empowerment	-0.035	0.032	0.26	-0.097	0.027
Gaming X Empowerment	0.024	0.037	0.51	-0.048	0.097
**Random effects**					
σ^2 between participants					
Intercept	5.701	.7649	.000	4.3832	7.4163
σ^2 within participants (error covariance structure) Heterogeneous 1st order autoregressive					

σ_1^2	3.691	.555	.000	2.748	4.959
σ_2^2	1.791	.455	.000	1.089	2.947
σ_3^2	.480	.243	.04	.177	1.296
ρ	.009	.153	.94	-.282	.300
**Model fit indices**					
-2LL	1753.649				
AIC	1841.649				

Table 4: Multilevel model for prescription medication overuse outcome measure

Parameter	Estimate	Std. Error	Sig.	95% Confidence Interval	
				Lower Bound	Upper Bound
**Fixed effects**					
Intercept	12.064	5.784	0.03	0.642	23.486
RA Duration	0.361	0.115	0.00	0.134	0.587
Age	-0.193	0.131	0.14	-0.452	0.067
Male	-2.962	3.057	0.33	-9.000	3.076
Elementary school	-6.062	7.679	0.43	-21.229	9.105
Middle school	2.782	4.886	0.57	-6.865	12.429
High school	-1.650	4.245	0.69	-10.028	6.728
Swiss	-5.510	3.416	0.10	-12.259	1.239
Working	-6.914	3.040	0.02	-12.916	-0.911
SocSup	7.748	4.963	0.12	-2.053	17.550
SocSupGaming	9.516	4.511	0.03	0.612	18.420
InfO	10.069	4.383	0.02	1.413	18.725
Gaming	4.310	4.864	0.37	-5.294	13.914
Time	-0.555	0.987	0.57	-2.509	1.400
RA Duration X Time	-0.038	0.019	0.04	-0.075	0.000
Age X Time	0.054	0.023	0.01	0.009	0.100
Male X Time	0.443	0.522	0.39	-0.591	1.476
Elementary school X Time	0.245	1.321	0.85	-2.369	2.859
Middle school X Time	-0.824	0.841	0.32	-2.489	0.840
High school X Time	-0.970	0.735	0.18	-2.425	0.484
Swiss X Time	0.197	0.583	0.73	-0.957	1.352
Working X Time	1.918	0.525	0.00	0.879	2.957
SocSup X Time	-1.613	0.836	0.056	-3.268	0.043
SocSupGaming X Time	-0.752	0.741	0.31	-2.219	0.715
InfO X Time	-0.109	0.740	0.88	-1.573	1.356
Gaming X Time	-1.050	0.806	0.19	-2.647	0.547
Empowerment	-0.340	0.238	0.15	-0.808	0.128

RA Duration X Empowerment	0.006	0.004	0.10	-0.001	0.014
Age X Empowerment	0.001	0.005	0.83	-0.008	0.011
Male X Empowerment	0.037	0.102	0.71	-0.163	0.238
Elementary school X Empowerment	0.485	0.284	0.08	-0.074	1.043
Middle school X Empowerment	0.120	0.204	0.55	-0.282	0.522
High school X Empowerment	0.150	0.188	0.42	-0.219	0.518
Swiss X Empowerment	0.087	0.114	0.44	-0.138	0.312
Working X Empowerment	0.117	0.109	0.28	-0.097	0.332
SocSup X Empowerment	-0.052	0.180	0.77	-0.405	0.302
SocSupGaming X Empowerment	0.001	0.157	0.99	-0.309	0.311
InfO X Empowerment	-0.139	0.148	0.34	-0.430	0.152
Gaming X Empowerment	0.074	0.175	0.67	-0.270	0.418
**Random effects**					
σ^2 between participants					
Intercept	102.351	13.665	.000	78.7850	132.9665
σ^2 within participants (error covariance structure) Heterogeneous 1st order autoregressive					
σ_1^2	105.819	15.718	.000	79.090	141.580
σ_2^2	77.688	13.273	.000	55.580	108.590
σ_3^2	2.626	5.319	.62	.049	139.158
ρ	-.141	.117	.22	-.361	.092
**Model fit indices**					
-2LL	3102.279				
AIC	3190.279				

Secondary Outcomes

Table 5: Multilevel model for empowerment measure

Parameter	Estimate	Std. Error	Sig.	95% Confidence Interval	
				Lower Bound	Upper Bound
**Fixed effects**					
Intercept	51.560	7.131	.000	37.493	65.627
RA Duration	-0.240	0.135	0.07	-0.507	0.027
Age	0.394	0.160	0.01	0.079	0.708
Male	2.331	3.820	0.54	-5.205	9.868
Elementary school	-12.432	9.533	0.19	-31.239	6.375
Middle school	-3.322	5.918	0.57	-14.996	8.351
High school	-10.095	5.105	0.04	-20.165	-0.025
Swiss	0.926	4.281	0.82	-7.520	9.372
Working	8.062	3.721	0.03	0.721	15.403
SocSup	-2.677	6.117	0.66	-14.744	9.390
SocSupGaming	3.674	5.432	0.5	-7.042	14.390
InfO	0.287	5.409	0.95	-10.384	10.958
Gaming	-1.449	5.981	0.80	-13.247	10.350
Time	-2.112	1.417	0.13	-4.915	0.690
RA Duration X Time	0.028	0.027	0.29	-0.025	0.081
Age X Time	0.029	0.033	0.37	-0.035	0.093
Male X Time	-1.659	0.753	0.02	-3.148	-0.170
Elementary school X Time	2.820	1.862	0.13	-0.865	6.505
Middle school X Time	0.121	1.196	0.92	-2.244	2.485
High school X Time	1.400	1.041	0.18	-0.659	3.459
Swiss X Time	0.864	0.843	0.30	-0.805	2.533
Working X Time	0.183	0.754	0.80	-1.308	1.674
SocSup X Time	2.591	1.215	0.03	0.187	4.995
SocSupGaming X Time	0.395	1.070	0.71	-1.721	2.512
InfO X Time	1.802	1.061	0.09	-0.298	3.902
Gaming X Time	2.291	1.172	0.05	-0.027	4.610
**Random effects**					
σ^2 between participants					
Intercept	194.933	26.713	.000	149.0182	254.994
σ^2 within participants (error covariance structure) Heterogeneous 1st order autoregressive					
σ_1^2	134.665	20.337	.000	100.162	181.054

σ_2^2	52.366	21.239	.01	23.648	115.955
σ_3^2	50.970	11.160	.000	33.184	78.289
ρ	-.155	.180	.38	-.477	.202
**Model fit indices**					
-2LL	3311.290				
AIC	3373.290				

Table 6: Multilevel model for rheumatoid arthritis knowledge measure

Parameter	Estimate	Std. Error	Sig.	95% Confidence Interval	
				Lower Bound	Upper Bound
**Fixed effects**					
Intercept	5.891	0.863	.000	4.186	7.596
RA Duration	0.011	0.016	0.52	-0.022	0.043
Age	-0.002	0.019	0.91	-0.040	0.036
Male	-0.171	0.465	0.71	-1.091	0.749
Elementary school	-0.044	1.112	0.96	-2.242	2.154
Middle school	0.416	0.720	0.56	-1.007	1.840
High school	1.130	0.621	0.07	-0.097	2.357
Swiss	-0.103	0.520	0.84	-1.130	0.925
Working	-0.161	0.453	0.72	-1.056	0.734
SocSup	1.317	0.739	0.07	-0.143	2.777
SocSupGaming	0.619	0.655	0.34	-0.676	1.913
InfO	0.563	0.652	0.38	-0.726	1.852
Gaming	1.286	0.722	0.07	-0.141	2.713
Time	0.298	0.196	0.13	-0.089	0.685
RA Duration X Time	-0.004	0.004	0.23	-0.012	0.003
Age X Time	-0.010	0.005	0.02	-0.019	-0.001
Male X Time	0.012	0.105	0.90	-0.195	0.220
Elementary school X Time	-0.253	0.250	0.31	-0.747	0.240
Middle school X Time	-0.120	0.166	0.47	-0.447	0.208
High school X Time	-0.156	0.144	0.28	-0.440	0.129
Swiss X Time	0.049	0.117	0.67	-0.183	0.281
Working X Time	-0.089	0.104	0.39	-0.295	0.118
SocSup X Time	-0.008	0.168	0.96	-0.340	0.323
SocSupGaming X Time	-0.064	0.147	0.66	-0.356	0.227
InfO X Time	0.082	0.146	0.57	-0.207	0.372
Gaming X Time	0.004	0.162	0.97	-0.316	0.325
**Random effects**					
σ^2 between participants					

Intercept	1.563	0.625	0.01	0.714	3.422
σ^2 within participants (error covariance structure) Heterogeneous 1st order autoregressive					
σ_1^2	2.585	0.525	0.000	1.736	3.847
σ_2^2	2.634	0.836	0.002	1.415	4.905
σ_3^2	1.452	0.577	0.01	0.667	3.163
ρ	0.445	0.154	0.004	0.101	0.694
**Model fit indices**					
-2LL	1606.662				
AIC	1668.662				
