# Supplementary material for: The Effect of Social Support Features and Gamification on a Web-Based Intervention for Rheumatoid Arthritis Patients: Randomized Controlled Trial
Source: J Med Internet Res. 2015 Jan 9;17(1):e14. doi: 10.2196/jmir.3510 (PMC4296094; doi:10.2196/jmir.3510)
Supplement: Supplementary file 5 [file jmir_v17i1e14_app5.pdf]

CONSORT-EHEALTH Checklist V1.6.2 Report

(based on CONSORT-EHEALTH V1.6), available at [http://tinyurl.com/consort-ehealth-v1-6].

3510

Date completed

5/3/2014 11:29:37

by

Ahmed Allam

Do social support features and gamification affect goal attainment of health websites? A randomized controlled trial of a web-based intervention for rheumatoid arthritis patients

TITLE

1a-i) Identify the mode of delivery in the title

The study was a web-based intervention and it is clearly mentioned in the title "[...] A randomized controlled trial of a web-based intervention for rheumatoid arthritis patients"

1a-ii) Non-web-based components or important co-interventions in title

item 1a-ii is not applicable since the intervention was all about manipulating the access to sections and features offered by a website.

1a-iii) Primary condition or target group in the title

The title mentions clearly the target group "[...] A randomized controlled trial of a web-based intervention for rheumatoid arthritis patients"

ABSTRACT

1b-i) Key features/functionalities/components of the intervention and comparator in the METHODS section of the ABSTRACT

The abstract includes a description about the type of manipulation "Objectives: The aim was to look into the effects of a Web-based intervention that included online social support features and gamification on physical activity, health care utilization, medication overuse, empowerment and RA knowledge of RA patients. The effect of gamification on website use was also investigated.

Methods: We conducted a 5-arm parallel randomized controlled trial for RA patients in Ticino (Italian-speaking part of Switzerland). The 157 recruited patients through brochures left with physician were randomly allocated to one of the 4 experimental conditions corresponding to different types of access to online social support and gamification features and a control group that had no access to the website. Data were collected at 3 time points through online questionnaires and a paper-pencil version for the control group at baseline, posttest 2 months later and a follow-up after another 2 months. "

1b-ii) Level of human involvement in the METHODS section of the ABSTRACT

There was no direct physician/therapist involvement (face-to-face or by telephone) during the intervention. The intervention included as part of the experimental manipulation pre-scheduled chat-room sessions that were moderated by the research team and involved the synchronous interaction between the patients and the doctors through the website.

1b-iii) Open vs. closed, web-based (self-assessment) vs. face-to-face assessments in the METHODS section of the ABSTRACT

"The 157 recruited patients through brochures left with physician were randomly allocated to one of the 4 experimental conditions corresponding to different types of access to online social support and gamification features and a control group that had no access to the website. Data were collected at 3 time points through online questionnaires and a paper-pencil version for the control group at baseline, posttest 2 months later and a follow-up after another 2 months. "

1b-iv) RESULTS section in abstract must contain use data

The results section included the analysis of the RCT and reported the estimated parameters with their corresponding p-values. "The best-fit multilevel models (growth curve models) that described the change in the primary outcomes over the course of the intervention included time and empowerment as time-variant predictors. The growth curve analyses of experimental conditions were always in comparison with the control group. Physical activity (amount spent on exercise) increased over time for patients having access to social support sections and gaming (unstandardized beta coefficient [B] =3.39, P=.02). The rate of change in health care utilization showed a significant decrease for patients accessing social support features (B=-.41, P=.01) and patients accessing both social support features and gaming (B=-.33, P=.03). Moreover, patients who had access to social support features witnessed a significant decrease rate of medication overuse (B=-1.61, P=.05). Patients who had access to either social support sections or the gaming experience of the website gained more empowerment (B=2.59, P=.03) (B=2.29, P=.05) respectively. Lastly, patients who were offered gamified experience used the website more often than the ones who were deprived from (t(91) = -2.27 P=.02) (U=812, P=.02). "

1b-v) CONCLUSIONS/DISCUSSION in abstract for negative trials

The intervention did not have negative consequences or effects on patients. Therefore item 1b-v is not applicable.

INTRODUCTION

2a-i) Problem and the type of system/solution

"The intervention used in this study is “ONESELF”, a Web-based intervention designed and operated for chronically ill patients with rheumatoid arthritis (RA). RA is a chronic systemic disease that affects the joints, connective tissues, muscles, tendons, and fibrous tissue [40]. Adults between the ages of 20-40 are predominantly targeted by the disease, which is more prevalent in women than in men [40]. Since, in terms of economics, RA affects people during an especially productive period of their lives, it is globally considered a serious public health problem. In Switzerland up to 1% of the population suffers from RA where its estimated cost on the Swiss society reaches 23,982 euros per patient a year [41]. The impact of RA goes beyond physical and economic aspects; it affects patients psychologically and emotionally, making them suffer severe consequences and losses [42]. ONESELF has informational and online support features and a gamified user experience, but access to these features was manipulated for the different experimental groups. We developed ONESELF in collaboration with the doctors of the Swiss Rheumatology Association (www.oneself.ch). ONESELF started in 2004 (early prototype and development) and pursued the aim of helping patients with chronic low back pain and fibromyalgia syndrome (FMS) to enhance their self-management and better cope with their conditions [43-47]. "

2a-ii) Scientific background, rationale: What is known about the (type of) system

"The basic idea behind informative eHealth offers issued by official or medical institutions is to help patients to better cope with chronic conditions, primarily by providing correct and up-to-date information [1]. The goals can be detailed as, among others, knowledge gain, better health-related quality of life, behavior change beneficial to patients' health, and less unnecessary utilization of the health system. Besides the provision of information, the Internet has more to offer to attain these goals; in particular, health websites can be designed to provide social support to their users.
The potential of these sites can be reached only if users log in regularly, especially if the website is dynamic. In order to increase the use of websites and to create a bond between the user and the website, features of gamification have recently been added to systems and websites delivering services to Internet consumers [2-4]. This gives rise to the question of whether social support features and gamification elements affect the attainment of the goal of health websites, provided they do have effects. [...] Literature reviews as [22] and more recent ones like [23] investigated the evidence of an effect of online peer-to-peer interactions on users/patients and Internet support groups (ISGs) in the area of depressive symptoms. According to the older review [22], virtual communities cannot harm people; however there was no evidence of benefit either, which suggests more research to understand for whom and under which condition social support could work [22]. As for [23] there was little high-quality evidence dealing with the efficacy of ISGs on coping with depression, suggesting a high need for high-quality randomized controlled trials (RCTs) in this domain. [...] In another perspective, the concept of "gamification" emerged recently. It is described as the application of game design elements in a non-game context to motivate or influence participation [2][33-34] and sometimes also refers to designing new serious games [35]. Significant knowledge increase by, and high users' appreciation of, gamified applications are shown in studies on gamifying laboratory experience for undergraduate microbiology students [36], evaluating a 3D serious game for advanced life support retraining [37], and major incident triage training [38]. However, much of the evidence on an influence of gamification on people's mind and behavior is anecdotal, with only one very recent systematic literature review [39] as discussed in [4]. [...] Only one study [35] emerged from [39] that used validated psychometric measurements, and its context was health/exercise. [...] Given the dearth in hard evidence for the effect of online social support and particularly gamification of web-based health interventions on health outcomes, we decided to conduct an experimental study that included both. The intervention used in this study is "ONESELF", a Web-based intervention designed and operated for chronically ill patients with rheumatoid arthritis (RA). [...] To our knowledge this is one of the first RCT studies [ISRCTN57366516] that includes gamification as part of experimental manipulation and studies its effect on cognitive and behavioral outcomes of RA-diagnosed patients. "

METHODS

3a) CONSORT: Description of trial design (such as parallel, factorial) including allocation ratio

"The main part of this study looks into the effects of social support features and gamification on the primary outcomes, expecting beneficial effects (more exercise, less healthcare utilization, less medication overuse) for the former, and treating the direction of an effect of gamification as an open research question. In a side analysis, the effect of gamification on the website use will be addressed."

3b) CONSORT: Important changes to methods after trial commencement (such as eligibility criteria), with reasons

No changes to the methods after trial and item 3b is not applicable.

3b-i) Bug fixes, Downtimes, Content Changes

There were no unexpected events during the intervention, the website and the manipulation worked well and there were no downtimes. The content was updated during the intervention especially the ones published by patients and doctors who were participating in the chatroom, forum and blog as part of the web-based intervention.

4a) CONSORT: Eligibility criteria for participants

"The inclusion criteria for patients were: 1 – to have received a diagnosis for RA from a doctor, 2 – to have cognitive function sufficient to use the website effectively, 3 - not to suffer from any other major chronic illness (e.g., cancer, diabetes), 4 – to have Internet access 5- willingness to use the website for at least 1 hour per week and 6 – to be fluent in the Italian language. "

4a-i) Computer / Internet literacy

There was no formal measure of computer literacy, however the inclusion criteria stated that patients need to 2 – to have cognitive function sufficient to use the website effectively 4 – to have Internet access 5- willingness to use the website for at least 1 hour per week

4a-ii) Open vs. closed, web-based vs. face-to-face assessments:

In the Procedure section of the manuscript: "Patients signed up for an account on the website by choosing a username and password. The first time patients signed in to the platform, they were prompted to fill in an online baseline questionnaire. Access to the website was blocked until the patients finished it. After that, patients were free to explore the different sections and features of the website available to the experimental group they belonged to. [...] Two months after the beginning of the intervention, the posttest was presented modally and patients had to complete it if they wished to continue using the site. The final follow up was filled in 2 months after the posttest. [...] The control group filled out a paper-pencil version, having been informed that we were interested in collecting general information about RA with the aim of developing an online platform that would be accessible to the public at a later date. "

In the Recruitment and participants section of the manuscript: "Recruitment lasted from November 2012 until February 2013. Patients were introduced to the experiment through brochures left with rheumatologists, physiotherapists, ergotherapists and psychologists. The patients were given the brochure with a brief description of the study and also a consent form to be signed and sent back to the research team (see Multimedia Appendix 2)."

4a-iii) Information giving during recruitment

"Recruitment lasted from November 2012 until February 2013. Patients were introduced to the experiment through brochures left with rheumatologists, physiotherapists, ergotherapists and psychologists. The patients were given the brochure with a brief description of the study and also a consent form to be signed and sent back to the research team (see Multimedia Appendix 2). "

4b) CONSORT: Settings and locations where the data were collected

"The study was conducted as an RCT experiment with RA patients in Ticino (Italian-speaking part of Switzerland). Data were collected from the last week of February 2013 until July 2013.
A new independent section about RA was created and added to the other two sections of ONESELF, which incidentally were completely rewritten in Drupal [48] on this occasion. The experiment tested, in a repeated-measure design, the effect of website sections and features offering social support and a corresponding gaming experience against a merely informative version of the website."

4b-i) Report if outcomes were (self-)assessed through online questionnaires

"The first time patients signed in to the platform, they were prompted to fill in an online baseline questionnaire. Access to the website was blocked until the patients finished it. [...] Two months after the beginning of the intervention, the posttest was presented modally and patients had to complete it if they wished to continue using the site. The final follow up was filled in 2 months after the posttest. [...] The control group filled out a paper-pencil version, having been informed that we were interested in collecting general information about RA with the aim of developing an online platform that would be accessible to the public at a later date. "

4b-ii) Report how institutional affiliations are displayed

item 4b-ii is not important, however in the manuscript we added Multimedia Appendix 2 that contains how institutional affiliations were displayed on the brochure and the consent form.

5) CONSORT: Describe the interventions for each group with sufficient details to allow replication, including how and when they were actually administered

5-i) Mention names, credential, affiliations of the developers, sponsors, and owners

In the acknowledgment section we mentioned the sponsors and the supporters who helped in the study. Moreover, in the manuscript there is a conflict of interest section. And for more information about institutional and other affiliations that were visible to patients, refer to Multimedia Appendix 2.

5-ii) Describe the history/development process
The web-based intervention was a complete rebuilt where a rheumatoid arthritis section was added and the old sections and content of the ONESELF platform were ported to Drupal. The whole intervention was developed using Drupal content management system by building custom modules that handled the manipulation and the gamification mechanism.
"A new independent section about RA was created and added to the other two sections of ONESELF, which incidentally were completely rewritten in Drupal [48] on this occasion"
5-iii) Revisions and updating
The development was frozen during the trial but we kept updating the content where patients and or physician contributed through posting to the forum, blog and chat-room.
5-iv) Quality assurance methods
The website content was developed with the help of physicians who checked the validity of the content and after iterative process, the material was simplified to be understandable by laypersons. Moreover, the website confirms with the Health on the Net (HON) code of conduct. "We developed ONESELF in collaboration with the doctors of the Swiss Rheumatology Association (www.oneself.ch). [...] The ONESELF website is HON-certified and compliant with the Health on the Net Foundation guidelines. "
5-v) Ensure replicability by publishing the source code, and/or providing screenshots/screen-capture video, and/or providing flowcharts of the algorithms used
We prepared multiple video demos that describe the overall features of the RCT intervention (see Multimedia Appendix 1) The website is still operating and could be accessed through (www.oneself.ch). However, not all features are still available. "For additional information and presentation of different features and sections implemented in the platform, refer to Multimedia Appendix 1."
5-vi) Digital preservation
The website (www.oneself.ch) is still operating and will continue to operate. Moreover, the website was archived by webcite at: http://www.webcitation.org/6PIKVZPoo
5-vii) Access
Video demos could be found in Multimedia Appendix 2 that demonstrates multiple aspects of the intervention. The access to the website is free and they only had to register and sign up for an account to access it. "Patients signed up for an account on the website by choosing a username and password. The first time patients signed in to the platform, they were prompted to fill in an online baseline questionnaire. Access to the website was blocked until the patients finished it. After that, patients were free to explore the different sections and features of the website available to the experimental group they belonged to."
5-viii) Mode of delivery, features/functionalities/components of the intervention and comparator, and the theoretical framework
"Patients were randomly allocated to one of four experimental groups or a control group (CG) with no access to the website. The randomization of the patients was based on a computerized random number generator that handled the patients' assignment to different groups.
One group of patients had access to informational sections only (InfO), another group additionally to social support sections but not to the gaming (SocSup), while a third group had access to the gaming but not the social support sections (Gaming) and a final group had access to everything (SocSupGaming). " Additionally, a whole section under "Website Sections and Features" was added in the manuscript describing in details the different sections and features that were available during the intervention.
5-ix) Describe use parameters
"For testing the effect of gamification, the usage of the website was indicated by the sum of the logged visits to each of the Sections 1-6 of ONESELF, which were registered in the access log table of the website's database. When a section was entered, a new visit was counted. In order to look at the effect of gamification on usage we grouped patients who had access to gaming (SocSupGaming and Gaming) and those who had not (SocSup and InfO). For comparison, t-test and Mann-Whitney U test were employed."
5-x) Clarify the level of human involvement
Some physicians were available to respond to the posts on the forum section and others participated in the pre-scheduled chat-room sessions.
"5.A forum and chat-room were implemented and made available to the patients. During the course of the intervention, 9 pre-scheduled sessions were offered in the chat-room. Patients were able to see the agenda of the planned sessions and the topic that would be tackled by each doctor. In each session, a different doctor participated in the chat with the patients, moderated by the research team. Patients discussed their questions and concerns with the doctor. The whole discussion was visible to all participants in the chat-room."
5-xi) Report any prompts/reminders used
"Moreover, a ticker was displayed on the different sections of the website showing the latest messages addressing the patients. In addition to sending emails, patients who had access to social support features were also notified by SMS about the chat-room appointments, inviting them to participate.
[...] A maximum of 2 phone calls were done as reminder to fill in the questionnaires. "
5-xii) Describe any co-interventions (incl. training/support)
There were no training/support sessions. item 5-xii is not applicable.
6a) CONSORT: Completely defined pre-specified primary and secondary outcome measures, including how and when they were assessed
There were primary and secondary outcome measures. Each described in subsection under the "Methods" and "Results" section in the manuscript.
"Participants filled in questionnaires at three occasions, baseline, posttest 2 months later and a follow-up after another 2 months."
6a-i) Online questionnaires: describe if they were validated for online use and apply CHERRIES items to describe how the questionnaires were designed/deployed
The questionnaires were pretested for face and content validity where reliable and valid scales were used as described in the "Measures" section of the manuscript.
6a-ii) Describe whether and how "use" (including intensity of use/dosage) was defined/measured/monitored
see subitem 5-ix above.
6a-iii) Describe whether, how, and when qualitative feedback from participants was obtained
Feedback could be obtained through email or by contacting the team through the contact form found on the website. Additionally, the forum and or chat-room session were a possible channel to get additional feedback.
6b) CONSORT: Any changes to trial outcomes after the trial commenced, with reasons
There were no changes to trial outcomes after the trial commenced. subitem 6b is not applicable.
7a) CONSORT: How sample size was determined
7a-i) Describe whether and how expected attrition was taken into account when calculating the sample size
We did not provide sample size or power calculations since no consensus could be found for calculating sample size for multilevel models. However according to (Maas and Hox, 2005) a sample size of 50 participants at level-2 is valid enough to provide reliable estimates. In addition we had 155 patients who were measured for three time points resulting in 465 data points that would suffice most of classical statistical tests (i.e. ANOVA, ANCOVA)
7b) CONSORT: When applicable, explanation of any interim analyses and stopping guidelines
No interim analysis occurred. subitem 7b is not applicable.
8a) CONSORT: Method used to generate the random allocation sequence

In the "Methods" section of the manuscript: "Participants were randomly allocated to the parallel experimental groups, unaware of any manipulation, blinded to each other. Each group had access to different sections and features of the website, including a control group with no access at all."

In the "Procedure" section: "Patients were randomly allocated to one of four experimental groups or a control group (CG) with no access to the website. The randomization of the patients was based on a computerized random number generator that handled the patients' assignment to different groups."

8b) CONSORT: Type of randomisation; details of any restriction (such as blocking and block size)

The randomization was based on simple randomization "Patients were randomly allocated to one of four experimental groups or a control group (CG) with no access to the website. The randomization of the patients was based on a computerized random number generator that handled the patients' assignment to different groups."

9) CONSORT: Mechanism used to implement the random allocation sequence (such as sequentially numbered containers), describing any steps taken to conceal the sequence until interventions were assigned

"The randomization of the patients was based on a computerized random number generator that handled the patients' assignment to different groups"

10) CONSORT: Who generated the random allocation sequence, who enrolled participants, and who assigned participants to interventions

see subitem 9 above.

11a) CONSORT: Blinding - If done, who was blinded after assignment to interventions (for example, participants, care providers, those assessing outcomes) and how

11a-i) Specify who was blinded, and who wasn't

The experiment was single blinded where patients were not aware of the manipulation and were blinded to each other. Additionally, the doctors/physicians did not know the details of the intervention or the different types of access of each group. They only knew the general goal of the experiment that is to test the effect of using the website by comparing patients who will have access to it and the ones who will not (control group).

11a-ii) Discuss e.g., whether participants knew which intervention was the "intervention of interest" and which one was the "comparator"

Patients were blinded to each other and they were not aware of the manipulation. see subitem 11a-i above.

11b) CONSORT: If relevant, description of the similarity of interventions

"Patients were randomly allocated to one of four experimental groups or a control group (CG) with no access to the website. [...] One group of patients had access to informational sections only (InfO), another group additionally to social support sections but not to the gaming (SocSup), while a third group had access to the gaming but not the social support sections (Gaming) and a final group had access to everything (SocSupGaming)."

12a) CONSORT: Statistical methods used to compare groups for primary and secondary outcomes

We used multilevel linear modeling for the primary and the secondary outcomes. "Data were analyzed using a multilevel linear modeling technique where the three measurement occasions were on the first level and the patients on the second level. Multilevel modeling was chosen because it is very well suited for the analysis of repeated measurements allowing for correct inferences as standard errors of the estimated parameters will not be underestimated and consequently the risk of inflation of type I errors will be reduced. It is known to be robust for unbalanced and missing data [53][54][55]. This approach is known as growth curve modeling; it studies growth trajectories over time, examining the pattern of change and taking into account the correlation between the measurement occasions for each patient by considering random effects of the parameters and additionally estimating the error variance-covariance matrix of the repeated measurements. "

12a-i) Imputation techniques to deal with attrition / missing values

We did not use any of the imputation techniques since the attrition was very low 7.74% (12/155) and similarly there were few missing values. Additionally, we chose multilevel modeling that uses maximum likelihood estimation for calculating the parameters and it is known to be robust for unbalanced and missing data. "It is known to be robust for unbalanced and missing data [53][54][55]"

12b) CONSORT: Methods for additional analyses, such as subgroup analyses and adjusted analyses

No additional or subgroup analysis was performed. item 12b is not applicable.

RESULTS

13a) CONSORT: For each group, the numbers of participants who were randomly assigned, received intended treatment, and were analysed for the primary outcome

The manuscript includes CONSORT flow diagram (Figure 3) that reports all the subitem 13a required information.

13b) CONSORT: For each group, losses and exclusions after randomisation, together with reasons

The manuscript includes CONSORT flow diagram (Figure 3).

13b-i) Attrition diagram

see subitem 13b above.

14a) CONSORT: Dates defining the periods of recruitment and follow-up

"The study was conducted as an RCT experiment with RA patients in Ticino (Italian-speaking part of Switzerland). Data were collected from the last week of February 2013 until July 2013."
"Recruitment lasted from November 2012 until February 2013. "

14a-i) Indicate if critical "secular events" fell into the study period

Not applicable.

14b) CONSORT: Why the trial ended or was stopped (early)

The trial did not stop early. subitem 14b is not applicable.

15) CONSORT: A table showing baseline demographic and clinical characteristics for each group

The manuscript includes a table (Table 2) that reports the baseline demographic for each group.

15-i) Report demographics associated with digital divide issues

see subitem 15 above.

16a) CONSORT: For each group, number of participants (denominator) included in each analysis and whether the analysis was by original assigned groups

16-i) Report multiple "denominators" and provide definitions

It was reported whenever it was relevant and possible.

16-ii) Primary analysis should be intent-to-treat

We followed intent to treat analysis. "All outcomes were self-reported and measured at 3 time points. Intention to treat analysis was followed and multilevel linear mixed models were used to study the change of outcomes over time."

17a) CONSORT: For each primary and secondary outcome, results for each group, and the estimated effect size and its precision (such as 95% confidence interval)

The manuscript reports in Tables 3 and 4 the estimated parameters of the multilevel models. Additionally, Multimedia Appendix 3, reports in detail the Primary and secondary outcomes multilevel models (fixed and random-effect estimated parameters, error variance-covariance matrix, standard errors, p-values and 95% confidence intervals in addition to model fit measures).

17a-i) Presentation of process outcomes such as metrics of use and intensity of use

"On average, participants paid 44.05 visits to the various sections of ONESELF in the period of data collection. Participants offered the gaming experience on average paid 62.35 visits to the website (SocSupGaming and Gaming together), while participants not offered the gaming experience visited the site less frequently 26.15 visits on average. As a result, there was a significant difference between the groups (t(91) = -2.27 P=.02) (U=812, P=.02). This suggests that groups who were offered gamified experience used the website more often than groups denied this experience."

17b) CONSORT: For binary outcomes, presentation of both absolute and relative effect sizes is recommended

item 17b is not applicable.

18) CONSORT: Results of any other analyses performed, including subgroup analyses and adjusted analyses, distinguishing pre-specified from exploratory

subitem 18 is not applicable. We did not perform any subgroup analysis.

18-i) Subgroup analysis of comparing only users

see subitem 18 above.

19) CONSORT: All important harms or unintended effects in each group

There was no harm or unintended effects on any of the groups as it has been shown in the manuscript. subitem 19 is not relevant.

19-i) Include privacy breaches, technical problems

No privacy breaches or technical problems occurred during the intervention. subitem 19-i is not relevant.

19-ii) Include qualitative feedback from participants or observations from staff/researchers

We only conducted quantitative analysis that was reported in the manuscript. subitem 19-ii is not relevant.

DISCUSSION

20) CONSORT: Trial limitations, addressing sources of potential bias, imprecision, multiplicity of analyses

20-i) Typical limitations in ehealth trials

A limitation section is included in the manuscript elaborating on the limitations of the study.
"The study as many others on Web-based interventions, used self-reported measures to assess changes in behavioral and health outcomes. Maybe more objective measures could be utilized to study the efficacy of gamification and social support that would complement the results presented. Moreover a 4-month period might not be sufficient to explore the longitudinal effect of the experimental condition on the changes in measured outcomes. A longer period with more measurement points would result in more precise estimates of the effect of the experimental manipulation on the growth curves that might take different functional forms than the linear one used in this study."

21) CONSORT: Generalisability (external validity, applicability) of the trial findings

21-i) Generalizability to other populations

Concerning gamification and online social support that our experimental manipulation was based on showed that there are positive potentials and promising desirable effects of both of them on patients’ behavioral and health outcomes when included in an eHealth intervention. The manuscript discusses the results in details with respect to other eHealth interventions that were applied in different settings with different patients, etc. This would argue in favor of the generalizability of our obtained results in different setting.

21-ii) Discuss if there were elements in the RCT that would be different in a routine application setting

In a real setting, depending on the web-based platform, there might not be available physicians who are ready to respond to patients' posts on forum or spend some time in chat-room discussing problems and challenges related to the disease.

22) CONSORT: Interpretation consistent with results, balancing benefits and harms, and considering other relevant evidence

22-i) Restate study questions and summarize the answers suggested by the data, starting with primary outcomes and process outcomes (use)

The manuscript discusses the primary and the secondary outcomes and relates the results with the previous literature and eHealth interventions.
"The study included cognitive and behavioral measures. By using multilevel modeling and focusing on growth curve models, we wanted to look for systematic differences between experimental conditions in their growth trajectories and understand which combination of time-variant predictors would explain and fit best the growth trajectories of each of the conditions. In addition, we explored the effect of each of these time-variant predictors on the growth trajectories of each of the experimental groups always compared to the control group and controlling for patients’ socio-demographics."

22-ii) Highlight unanswered new questions, suggest future research

"[...] the study pointed to the positive potentials and the promising desirable effects of online social support and especially gamification on patients’ behavioral and health outcomes when included in an eHealth intervention. Hence, for the future, more research on the efficacy of gamification applied in different situations (patients diagnosed by different disease, different targeted platforms i.e. mobile applications) is needed and to understand which gamification strategies that would help the most in benefiting the patients and meeting the objectives and goals of health related platforms."

Other information

23) CONSORT: Registration number and name of trial registry

"Trial Registration: International Standard Randomized Controlled Trial Number (ISRCTN): 57366516; http://www.controlled-trials.com/ISRCTN57366516 (Archived by webcite at http://www.webcitation.org/6PBvvAvvV)"

24) CONSORT: Where the full trial protocol can be accessed, if available

see subitem 23 above.

25) CONSORT: Sources of funding and other support (such as supply of drugs), role of funders

In the acknowledgement section of the manuscript we included our sources of funding and other supporting institutions that helped us during the intervention.

X26-i) Comment on ethics committee approval

"The study was approved by the Ethical Committee of Canton Ticino (the Italian-speaking part of Switzerland). "

x26-ii) Outline informed consent procedures

"The patients were given the brochure with a brief description of the study and also a consent form to be signed and sent back to the research team (see Multimedia Appendix 2)."

X26-iii) Safety and security procedures

There were no security threats and the privacy was a priority in our intervention.

X27-i) State the relation of the study team towards the system being evaluated

A conflicts of interest section is included in the manuscript.
